# Author Correction: CAR-T cell therapy-related cytokine release syndrome and therapeutic response is modulated by the gut microbiome in hematologic malignancies

**DOI:** 10.1038/s41467-024-47038-5

**Published:** 2024-03-27

**Authors:** Yongxian Hu, Jingjing Li, Fang Ni, Zhongli Yang, Xiaohua Gui, Zhiwei Bao, Houli Zhao, Guoqing Wei, Yiyun Wang, Mingming Zhang, Ruimin Hong, Linqin Wang, Wenjun Wu, Mohamad Mohty, Arnon Nagler, Alex H. Chang, Marcel R. M. van den Brink, Ming D. Li, He Huang

**Affiliations:** 1 Bone Marrow Transplantation Center, The First Affiliated Hospital, School of Medicine, Zhejiang University, Hangzhou, China; 2 Zhejiang Province Engineering Laboratory for Stem Cell and Immunity Therapy, Hangzhou, China; 3 Institute of Hematology, Zhejiang University, Hangzhou, China; 4 Zhejiang Laboratory for Systems & Precision Medicine, Zhejiang University Medical Center, Hangzhou, China; 5 State Key Laboratory for Diagnosis and Treatment of Infectious Diseases, National Clinical Research Center for Infectious Diseases, Collaborative Innovation Center for Diagnosis and Treatment of Infectious Diseases, The First Affiliated Hospital, Zhejiang University School of Medicine, Hangzhou, China; 6 Research Center for Air Pollution and Health, Zhejiang University, Hangzhou, China; 7 Department of Hematology, Sorbonne University, Hospital Saint Antoine, Paris, France; 8 INSERM UMRs 938, and EBMT Paris Study office/CEREST-TC, Paris, France; 9 Hematology and Bone Marrow Transplantation Division, Chaim Sheba Medical Center, Tel-Hashomer, Israel; 10 Clinical Translational Research Center, Shanghai Pulmonary Hospital, Tongji University School of Medicine, Shanghai, China; 11 Department of Immunology, Sloan Kettering Institute, Memorial Sloan Kettering Cancer Center, New York, NY USA

**Keywords:** Acute lymphocytic leukaemia, Myeloma, Immunization, Predictive markers

Correction to: *Nature Communications* 10.1038/s41467-022-32960-3, published online 09 September 2022

The original version of this Article had numerical mistakes in the source data corresponding to figure 1a, some values (or textual entries) were missing (cells 1P, 18P), and some were incorrect (cells 3Q, 4J, 11J-P, 17K-L, 18N, 30B, 30E, 30J-K, 30N, 39E, 39J, 40E, 40J, 41E, 54J, 63J-L, 65B, 65K, 65N, 73E, 73J, 74E, 74J, 78N-P, 85J). These are replaced with the correct entries.

Column B of the source data corresponding to Supplementary Table 1 is now deleted, due to confidentiality concerns.

Accordingly, the text of the “Clinical trial outcomes” section of the Results has been corrected:

Line 10: “Within 1 month after BCMA CAR-T cell infusion, 2 patients died of cerebral hemorrhage and 4 died of severe infections.

Of the 95 evaluable patients, 91 (95.8%) had an overall response.”

Line 13: “With a median follow-up time of 21.2 months (95% CI, 18.4–32.1), the median progression-free survival (PFS) was 12.0 (95% CI, 8.1–15.7) months. The 1-year OS and PFS rates were 0.70 (95% CI, 0.61–0.80) and 0.48 (95% CI, 0.39–0.59), respectively.

### Supplementary information

Updated Source Data

