# Supplementary material for: CAR-T cell therapy-related cytokine release syndrome and therapeutic response is modulated by the gut microbiome in hematologic malignancies
Source: Nat Commun. 2022 Sep 9;13:5313. doi: 10.1038/s41467-022-32960-3 (PMC9461447; doi:10.1038/s41467-022-32960-3)
Supplement: Supplementary file 1 — Supplementary Information [file 41467_2022_32960_MOESM1_ESM.pdf]

Supplementary Materials for

CAR-T cell therapy-related cytokine release syndrome and therapeutic response is modulated by the gut microbiome in hematologic malignancies

Yongxian Hu, Jingjing Li, Fang Ni, Zhongli Yang, Xiaohua Gui, Zhiwei Bao, Houli Zhao, Guoqing Wei, Yiyun Wang, Mingming Zhang, Ruimin Hong, Linqin Wang, Wenjun Wu, Mohamad Mohty, Arnon Nagler, Alex H. Chang*, Marcel R. M. van den Brink*, Ming D. Li*, He Huang*

***Correspondence.** E-mail: huanghe@zju.edu.cn (H.H.); ml2km@zju.edu.cn (M.L.)

This PDF file includes:

Supplementary Figures:

Supplementary Fig. 1. CRS complication and decreased tumor burden after BCMA CAR-T cell therapy.

Supplementary Fig. 2. Clinical outcomes and CRS complications after CAR-T cell therapy in r/r B-ALL group.

Supplementary Fig. 3. Clinical outcomes and CRS complication after CAR-T cell therapy in r/r B-NHL group.

Supplementary Fig. 4. Changes of microbial composition across CAR-T therapy in NHL, ALL and MM patients.

Supplementary Fig. 5. Correlation of genera with clinical response in MM, NHL and ALL patients.

Supplementary Fig. 6. Correlation of PFS with genera Sutterella and Faecalibacterium in the discovery MM cohort.

Supplementary Fig. 7. Temporal differences between patients with severe and mild CRS in the discovery MM cohort.

Supplementary Fig. 8. Temporal differences between patients with severe, and moderate CRS in the discovery MM cohort.

Supplementary Fig. 9. Differences of bacteria abundances between patients with different CRS levels in the 38 MM validation cohort.

Supplementary Fig. 10. Differential KEGG pathways between CR and PR groups in the discovery MM cohort.

Supplementary Fig. 11. Differential KEGG pathways between CRS groups in the discovery MM cohort.

Supplementary Fig. 12. Differential analysis of KEGG pathways in the 38 MM validation cohort.

Supplementary Fig. 13. Differential metabolites between the CR and PR groups in the 38 MM validation cohort.

Supplementary Fig. 14. Differential metabolites between different CRS groups in the 38 MM validation cohort.

Supplementary Fig. 15. Pathway enrichment analysis for differentially abundant metabolites between CR and PR patients in 38 MM validation cohort.

Supplementary Fig. 16. Differences of immune cells and inflammatory markers between CR and PR groups in the discovery MM cohort.

Supplementary Fig. 17. Flow cytometry gate strategies.

Supplementary Tables:

Supplementary Table 1. Baseline characteristics of multiple myeloma patients included in final fecal microbiome analyses cohorts with different efficacy groups.

Supplementary Table 2. Baseline characteristics of multiple myeloma patients included in final fecal microbiome analyses cohorts with different CRS grade groups.

Supplementary Table 3. Demographic and clinical characteristics of B-ALL and B-NHL patients included in final fecal microbiome analyses cohorts.

Supplementary Methods

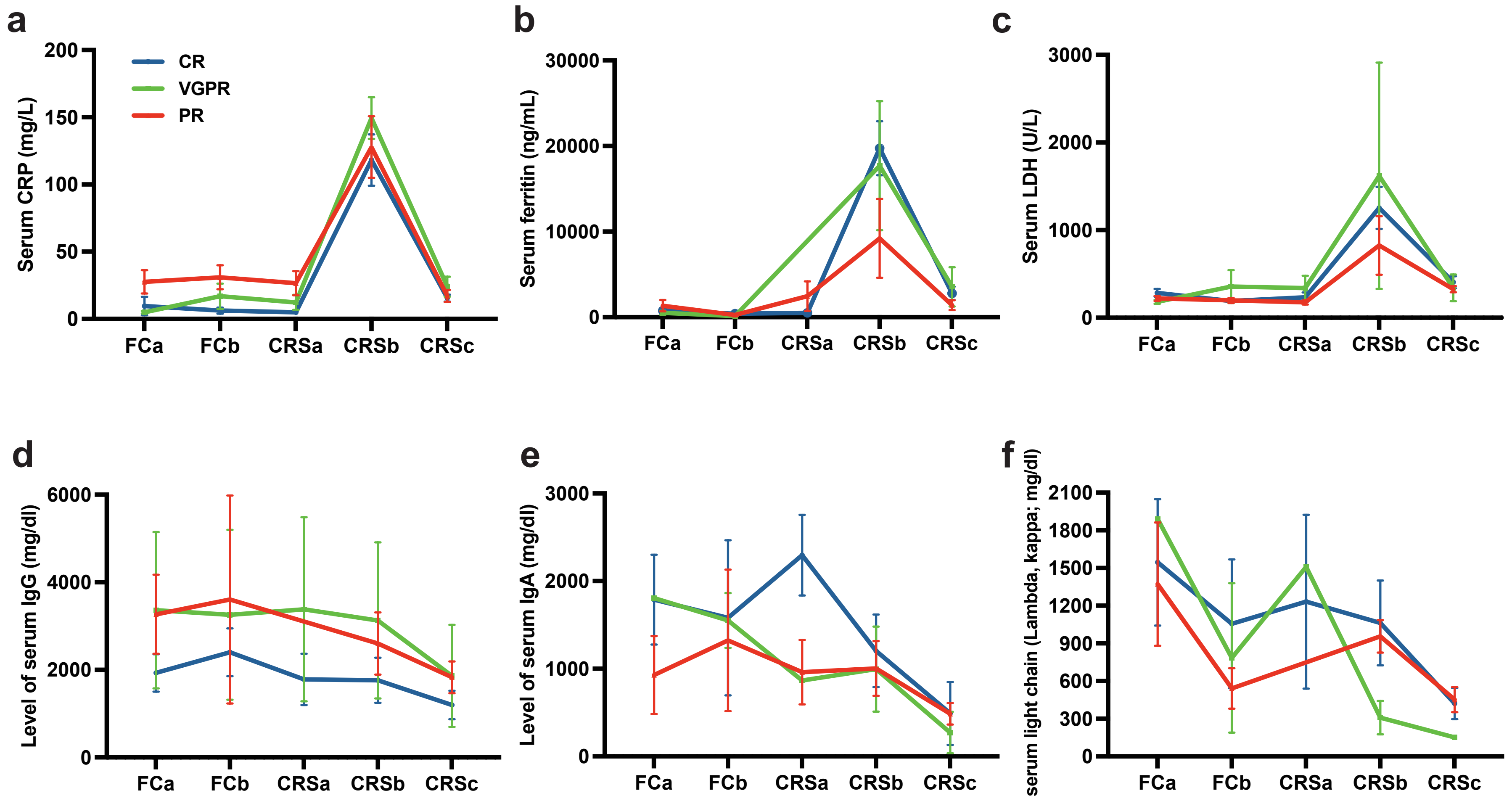

Supplementary Fig. 1. CRS complication and decreased tumor burden after BCMA CAR-T cell therapy. **(a-c)** Serum concentrations of C-reactive protein (CRP), ferritin, and lactic dehydrogenase (LDH) in different therapy stages among the CR, VGPR, and PR groups. **(d-f)** Changes in serum concentrations of immunoglobulin concentration (IgG, IgA) and κ and λ light chains in different therapy stages. CR group (n=24 biologically independent patients), VGPR group (n=6 biologically independent patients), and PR group (n=11 biologically independent patients). Data are presented as mean values +/- SEM.

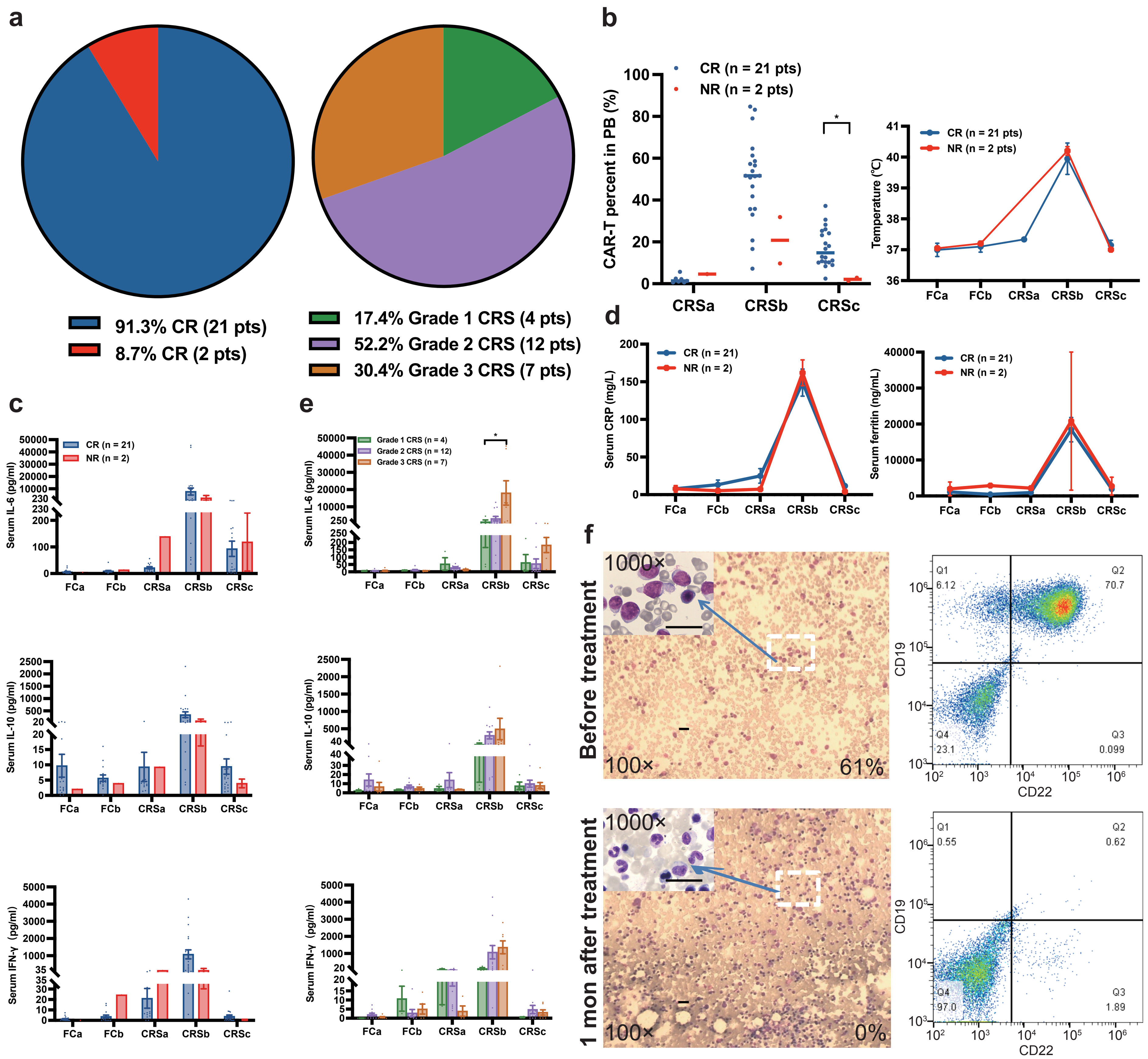

Supplementary Fig. 2. Clinical outcomes and CRS complications after CAR-T cell therapy in r/r B-ALL group. (a) Clinical response and CRS grade distribution in 23 r/r B-ALL patients. **(b-d)** Comparisons of CAR-T cell percentage in PB (assessed by FACS), body temperature, serum concentrations of IL-6, IL-10, IFN-γ, C-reactive protein (CRP), and ferritin between the CR (n=21 biologically independent patients) and NR (n=2 biologically independent patients) groups. Blue and red colors indicate CR and NR group, respectively. The data are presented as mean values +/- SEM. Significance determined by two-sided Mann Whitney test. * $p < 0.05$, ** $p < 0.01$. **(e)** Serum concentrations of IL-6, IL-10, and IFN-γ in different therapy stages between different CRS grade groups. (Grade 1 CRS group: n=4 biologically independent patients, Grade 2 CRS group: n=12 biologically independent patients, Grade 3 CRS group: n=7 biologically independent patients). Data are presented as mean values +/- SEM. Significance determined by two-sided Kruskal-Wallis test and adjustments were made for multiple comparison. * $p < 0.05$, ** $p < 0.01$. **(f)** More than half (61%) of the cells in representative r/r B-ALL patient's bone marrow were leukemia cells. After one month of CAR-T cell infusion, a complete absence of bone marrow leukemia cells was achieved, and leukemia cells were almost undetectable by flow cytometry. The bar indicates a length of 5μm.

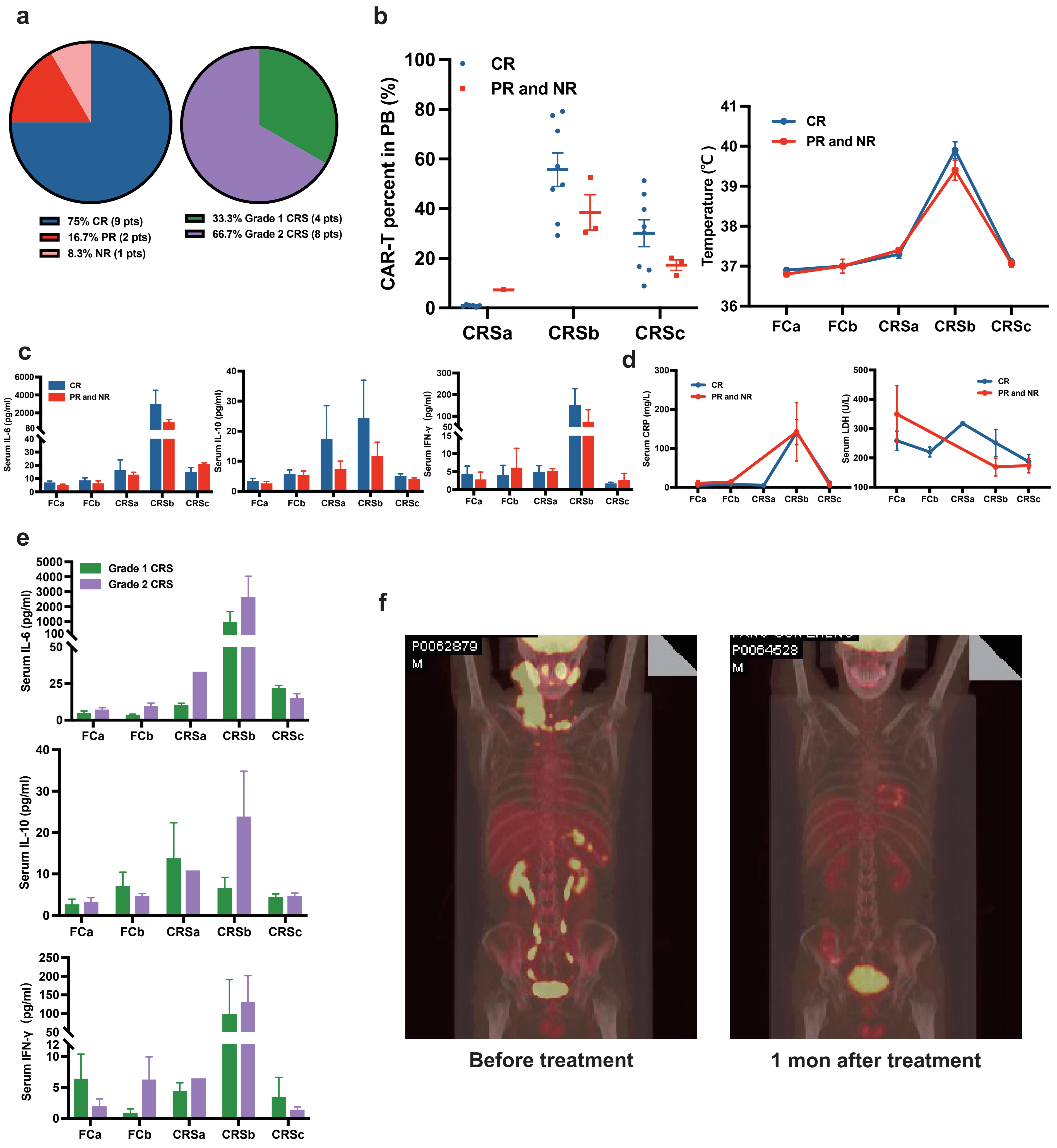

Supplementary Fig. 3. Clinical outcomes and CRS complication after CAR-T cell therapy in r/r B-NHL group. (a) Clinical response and CRS grade distribution in 12 r/r B-NHL patients. **(b-d)** CAR-T cell percentages in PB were detected continuously by FACS after infusion. Body temperature, serum concentrations of IL-6, IL-10, IFN-γ, C-reactive protein (CRP), and lactic dehydrogenase (LDH) in different therapy stages among the CR (n=9 biologically independent patients), PR (n=2 biologically independent patients), and NR groups (n=1 biologically independent patients). The data are presented as mean values+/- SEM. **(e)** Serum concentrations of IL-6, IL-10, and IFN-γ in different therapy stages among different CRS grades are presented. (Grade 1 CRS group: n=4 biologically independent patients, Grade 2 CRS group: n=8 biologically independent patients). The data are presented as mean values +/- SEM. **(f)** A representative r/r B-NHL patient with an

impressive antilymphoma response. The PET-CT scan showed almost complete elimination of extensive tumor cells after treatment.

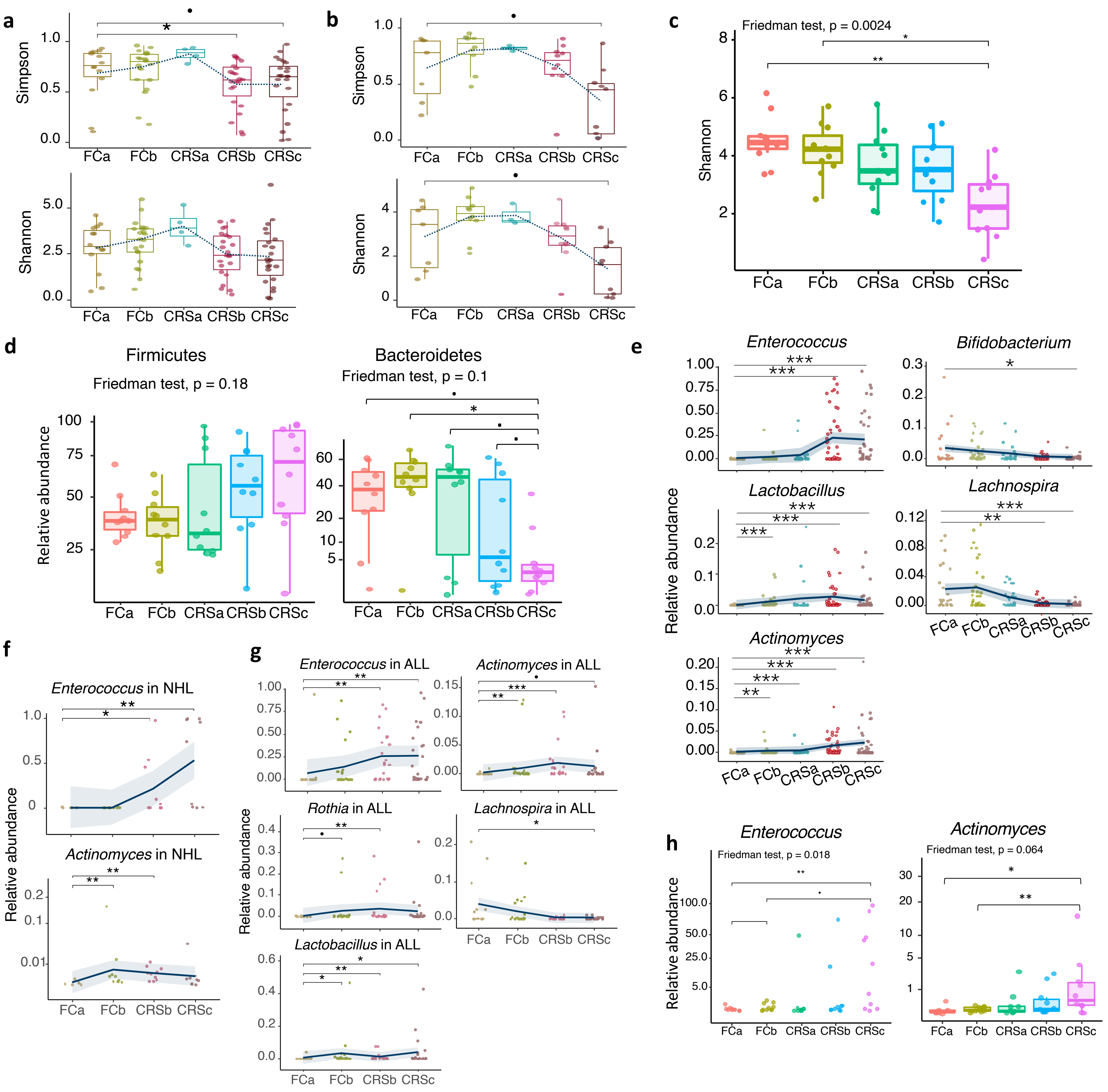

Supplementary Fig. 4. Changes of microbial composition across CAR-T therapy in NHL, ALL and MM patients. (a) Shannon and Simpson diversity indices of gut microbiome across CART therapy stages in all patients with acute lymphoblastic leukemia (ALL, n = 23). Simpson index: FCa versus CRSb, p = 0.036, FCa versus CRSc, p = 0.088. **(b)** Same as (A) for non-Hodgkin lymphoma (NHL) patients (n = 12). Simpson index: FCa versus CRSc, p = 0.071; Shannon index: FCa versus CRSc, p = 0.070. **(c)** Shannon diversity of the validation MM patients. Significance tested by Friedman's test and two-tailed Wilcoxon rank-sum test of pairwise combinations with multiple testing correction (n =10). FCa versus CRSc, p = 0.029; FCa versus CRSb, p = 0.02. **(d)** Relative abundance of phyla Firmicutes and Bacteroidetes across therapy stages in the validation MM cohort. Significance was assessed by Friedman's test and two-tailed Wilcoxon rank-sum test of pairwise combinations with multiple testing correction (n=10). Bacteroidetes: FCa versus CRSc, p = 0.091; FCb versus CRSc, p = 0.039; CRSa versus CRSc, p = 0.091; CRSb versus CRSc, p = 0.093. **(e)** Relative abundance of genus-level taxa enriched or depleted across therapy stages in the discovery myeloma cohort. Significance was assessed with two-tailed Wilcoxon rank-sum test (n = 43). Lines are presented as mean values ± SEM. Enterococcus: FCa versus CRSc, p = 2.8e-09; FCa versus CRSb, p = 1e-06. Bifidobacterium: FCa versus CRSc, p = 0.019. Lactobacillus: FCa versus CRSc, p = 0.00054; FCa versus

CRSb, p = 5.1e-05; FCa versus FCb, p = 0.00097. Lachnospira: FCa versus CRSc, p = 0.0003; FCa versus CRSb, p = 0.004. Actinomyces: FCa versus CRSc, p = 0.0004; FCa versus CRSb, p = 4.5e-06; FCa versus CRSa, p = 0.00024; FCa versus FCb, p = 0.001. **(f)** Same as **(e)** for NHL patients (n = 12). Enterococcus: FCa versus CRSc, p = 0.0029; FCa versus CRSb, p = 0.039. Actinomyces: FCa versus CRSb, p = 0.0021; FCa versus CRSb, p = 0.0072. **(g)** Same as **(e)** for ALL patients (n = 23). Enterococcus: FCa versus CRSc, p = 0.0016; FCa versus CRSb, p = 0.017. Actinomyces: FCa versus CRSc, p = 0.053; FCa versus CRSb, p = 0.00026; FCa versus FCb, p = 0.0063. Rothia: FCa versus CRSb, p = 0.0035; FCa versus FCb, p = 0.058. Lachnospira: FCa versus CRSc, p = 0.035. Lactobacillus: FCa versus CRSc, p = 0.011; FCa versus CRSb, p = 0.0044; FCa versus FCb, p = 0.047. **(h)** Relative abundance of genus *Enterococcus* and *Actinomycs* in the validation MM patients. Significance tested by Friedman's test and two-tailed Wilcoxon rank-sum test of pairwise combinations (n =10). FCa versus FCb, p = 0.027; FCa versus CRSc, p = 0.004; FCb versus CRSc, p = 0.014. In **a-d, h,** Boxplots indicate the median (thick bar), first and third quartiles (lower and upper bounds of the box, respectively), lowest and highest data value within 1.5 times the interquartile range (lower and upper bounds of the whisker). In a-h, ▪ p<0.1, * p < 0.05, ** p < 0.01, *** p < 0.001.

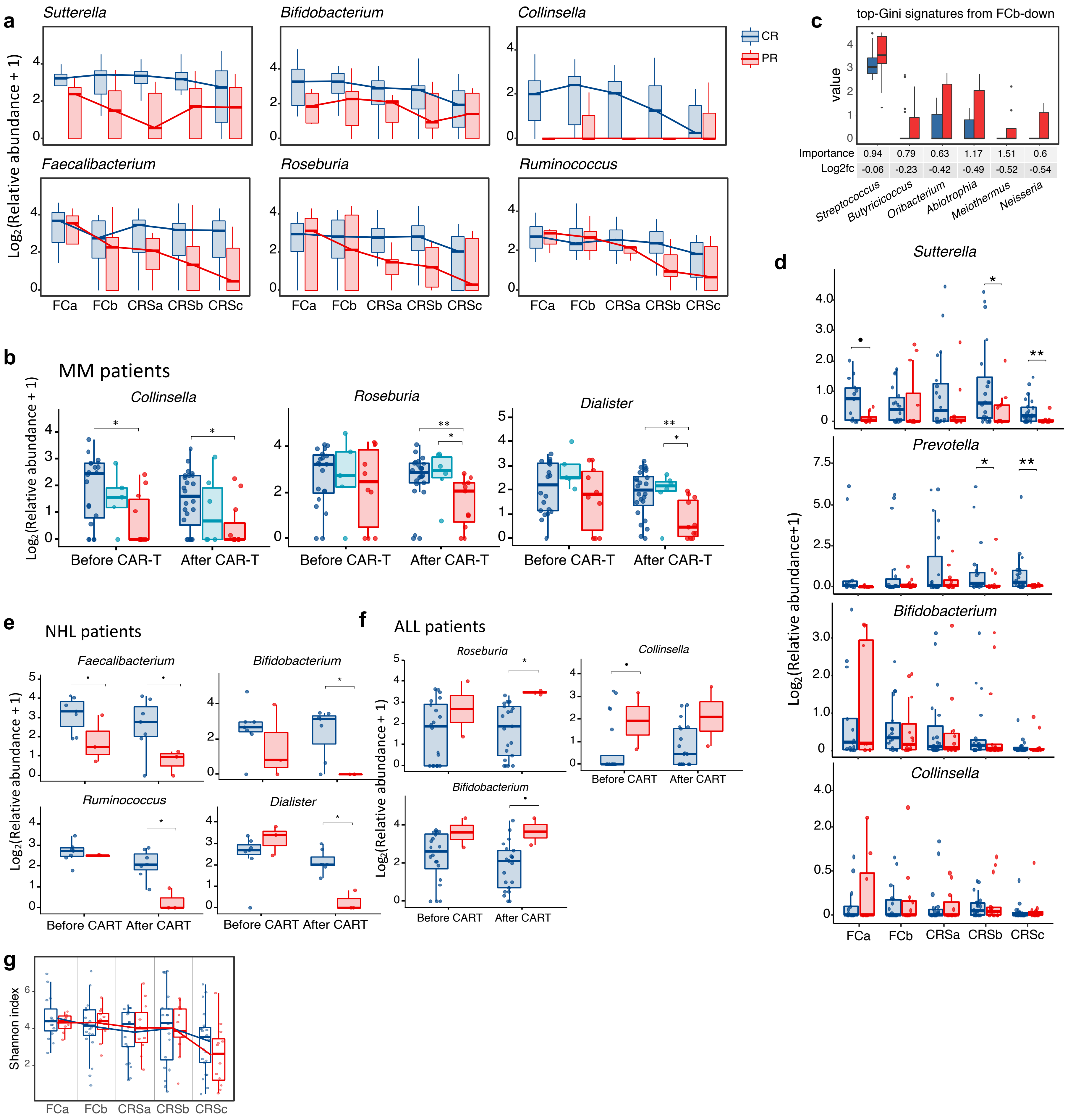

Supplementary Fig. 5. Correlation of genera with clinical response in MM, NHL and ALL patients. (a) Time-course differences in relative abundance (log transformed) of six representative genera between CR and PR groups in MM cohort (n = 35). **(b)** Average bacterial abundance of CR (blue), VGPR(cyan), and PR(red) of MM patients (n = 43) before and after CAR-T cell infusion. Significance tested by two-tailed Wilcoxon rank-sum test**.** Collinsella: before CAR-T, p = 0.011; after CAR-T, p = 0.019. Roseburia after CAR-T: CR versus PR, p = 0.0052; VGPR versus PR, p = 0.031. Dialister after CAR-T: CR versus PR, p = 0.04; VGPR versus PR, p = 0.026. **(c)** Relative abundance (log transformed) of top discriminative PR-enriched signatures at post-chemotherapy (FCb) timepoint identified by random forest (RF) feature selection procedure. **(d)** Relative abundance of genus *Sutterella*, *Prevotella*, *Collinsella* and *Bifidobacterium* bewteen the CR (blue) and non-CR (red) patients in the 38 validation MM cohort (n = 38). Significance tested by two-tailed Wilcoxon rank-sum test**.** Sutterella: FCa, p

= 0.081; CRSb, p.= 0.081; CRSc, p = 0.027. Prevotella: CRSb, p = 0.044; CRSc, p = 0.01. **(e)** Mean bacterial abundance before and after CAR-T cell infusion in non-Hodgkin's lymphoma (NHL) patients (n = 12; CR: blue; non-CR: red). Significance tested by two-tailed Wilcoxon rank-sum test**.** Faecalibacterium: before CAR-T, p = 0.067; after CAR-T, p = 0.086. Bifidobacterium: after CAR-T, p = 0.046. Ruminococcus: after CAR-T, p = 0.04. Dialister: after CAR-T, p = 0.022. **(f)** Mean bacterial abundance before and after CAR-T cell infusion in acute lymphoblastic leukemia (ALL) patients (n = 23; CR: blue; non-CR: red). Significance tested by two-tailed Wilcoxon rank-sum test**.** Roseburia: after CAR-T, p = 0.025. Collinsella: before CAR-T, p = 0.062. Bifidobacterium: after CAR-T, p = 0.072. **(g)** Differences of Shannon diversity between CR (blue) and non-CR (red) subjects in the 38 MM validation cohort (n = 38). Red and blue color indicate PR and CR, respectively. In b, d-f, significance was assessed by two-tailed Wilcoxon rank-sum test. • p < 0.1, * p < 0.05, ** p < 0.01. In a-f, the values of bacteria abundances were log2 transformed relative abundance in percentage with a pseudo 1 added, that is log2(Percentage+1). Boxplots indicate the median (thick bar), first and third quartiles (lower and upper bounds of the box, respectively), lowest and highest data value within 1.5 times the interquartile range (lower and upper bounds of the whisker).

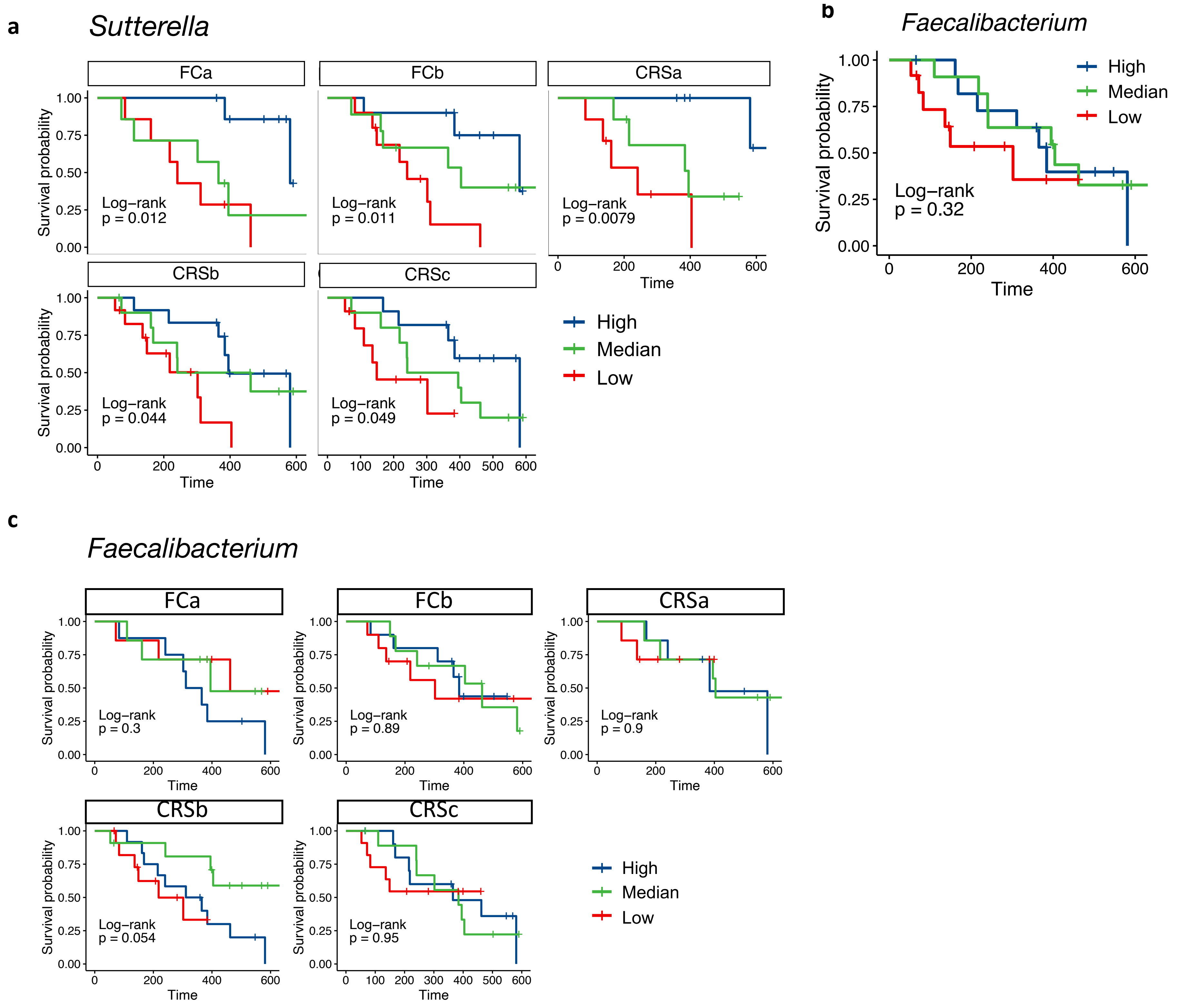

Supplementary Fig. 6. Correlation of PFS with genera *Sutterella* and *Faecalibacterium* in the discovery MM cohort. **(a)** Kaplan-Meier (KM) plot of PFS curves by log-rank test in patients with high (dark blue), median (green), or low (red) abundance of *Sutterella*. The PFS curves for different therapy stages were plotted independently. **(b)** Kaplan-Meier plot of PFS curves by log-rank test for patients with high (dark blue), median (green), or low (red) abundance of *Faecalibacterium*. Abundance of genus *Faecalibacterium* was in terms of median abundance at all timepoints. **(c)** Same as (a) for *Faecalibacterium.*

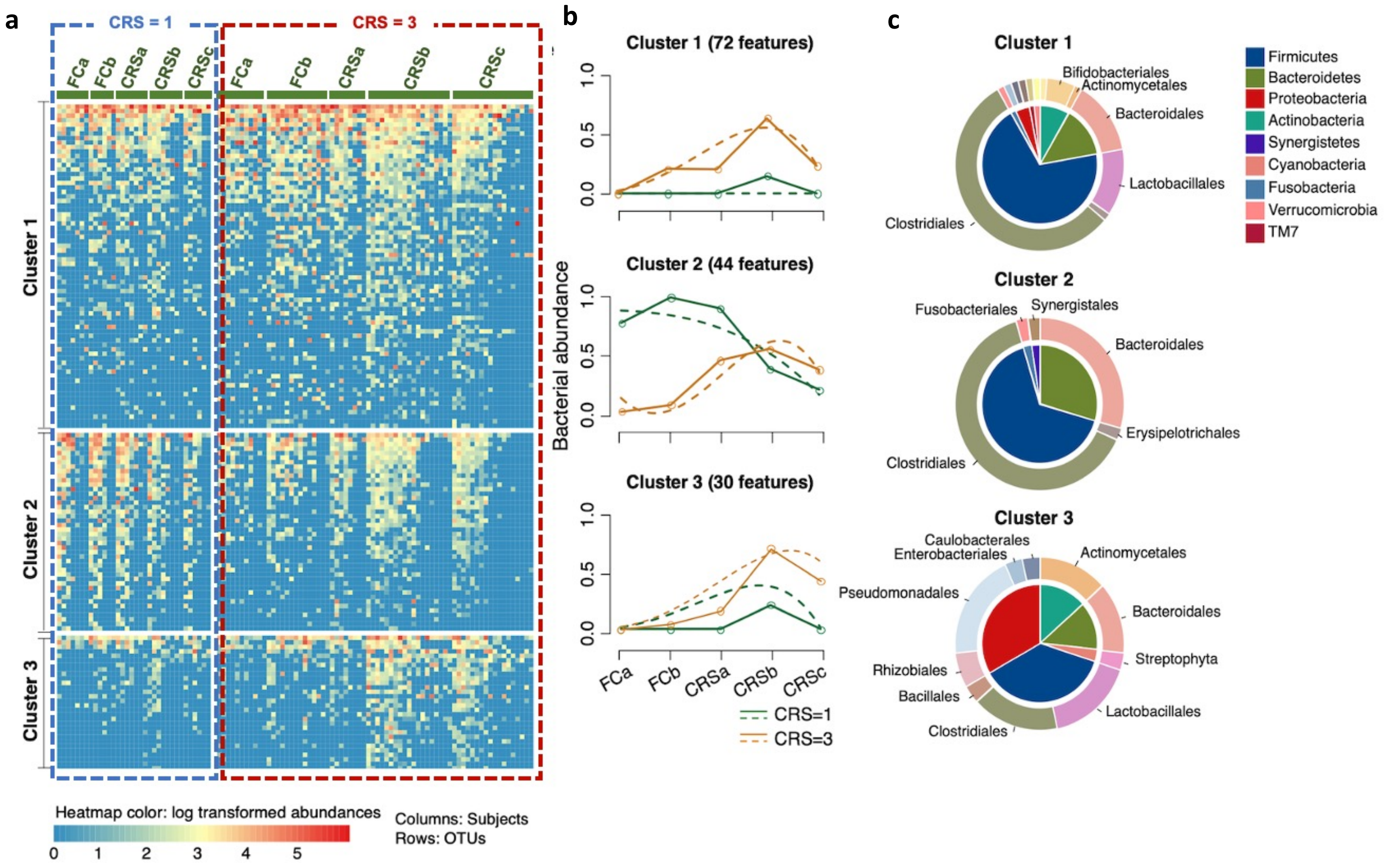

Supplementary Fig. 7. Temporal differences between patients with severe and mild CRS in the discovery MM cohort. **(a)** Heatmap for abundance of OTUs with significant (FDR < 0.05) temporal differences between severe (CRS = 3) and mild (CRS = 1) CRS groups. Rows denote bacterial OTUs grouped into three sets according to their abundance profile and then sorted by mean abundance within each set. Individual samples are organized in columns, with colored bars depicting their CRS grade groups and therapy stages. **(b)** Profiles of significant gene clusters correspond to (**a**). Solid lines denote median profile of abundance of OTUs within cluster for each experimental group through time. Fitted curve of each group is displayed as dotted line. **(c)** Phylogenetic composition of OTUs within each cluster in (a) at phylum and order levels.

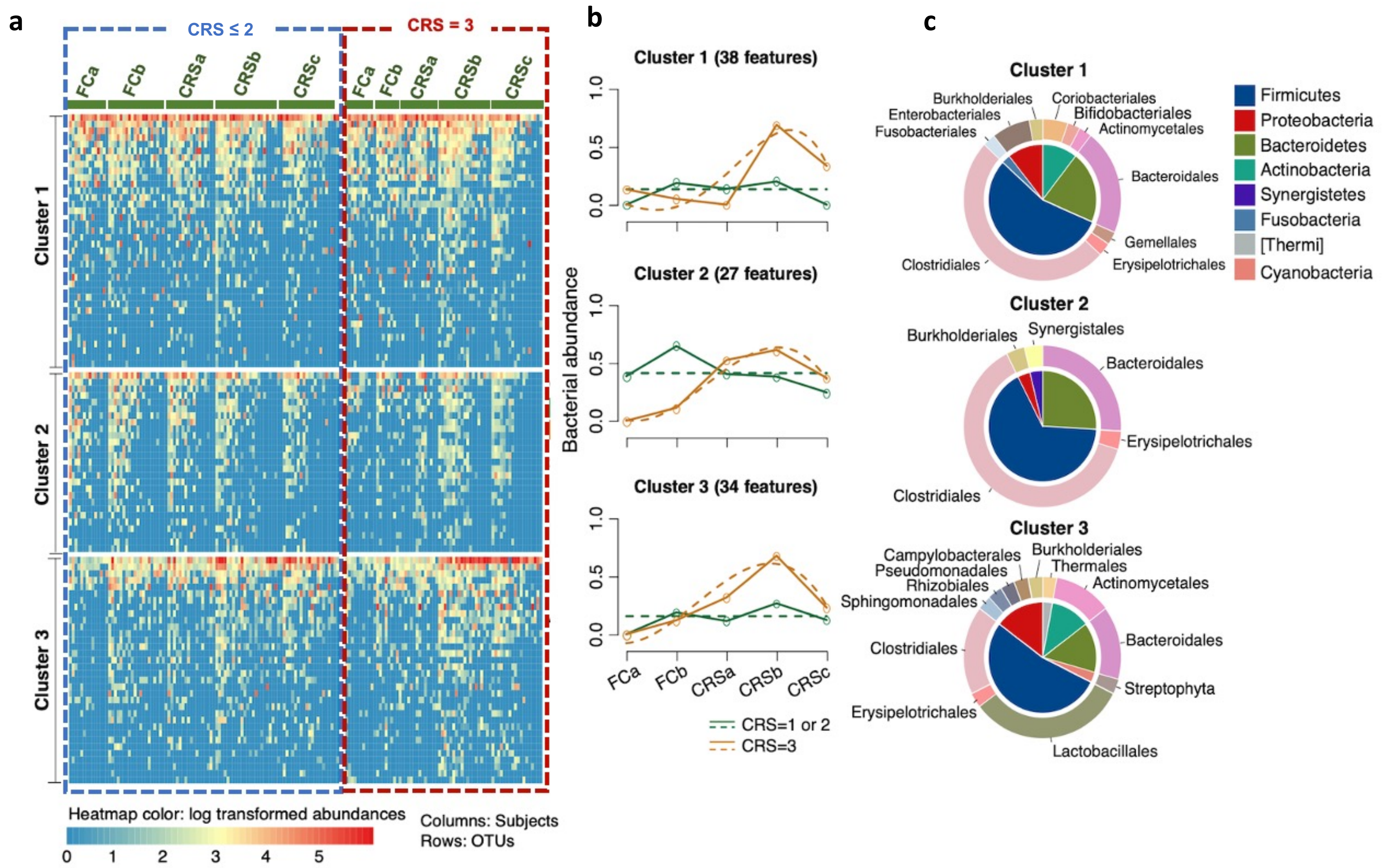

Supplementary Fig. 8. Temporal differences between patients with severe, and moderate CRS in the discovery MM cohort. (a) Heatmap for abundance of OTUs with significant (FDR < 0.05) temporal differences between severe (CRS = 3) and moderate (CRS ≤2) CRS groups. Rows denote bacterial OTUs grouped into three sets according to their abundance profile and then sorted by mean abundance within each set. Individual samples are organized in columns, with colored bars depicting their CRS grade groups and therapy stages. **(b)** Profiles of significant gene clusters correspond to (a). Solid lines denote the median profile of abundance of OTUs within cluster for each experimental group through time. Fitted curve of each group is displayed as dotted line. **(c)** Phylogenetic composition of OTUs within each cluster in (a) at phylum and order levels.

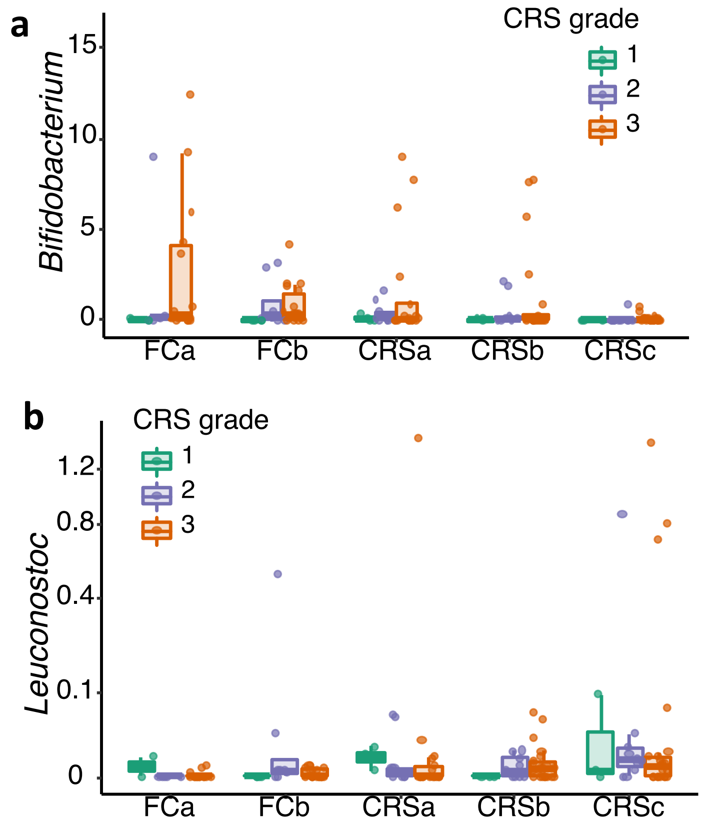

Supplementary Fig. 9. Differences of bacteria abundances between patients with different CRS levels in the 38 MM validation cohort. (a) Differences of genus *Bifidobacterium* between patients with different CRS levels at five timepoints in the 38 MM validation cohort (n = 38). **(b)** Differences of genus *Leuconostoc* between patients with different CRS levels at five timepoints in the 38 MM validation cohort. Significance was assessed with Wilcoxon rank-sum test. No significant difference was observed (n = 38). Boxplots indicate the median (thick bar), first and third quartiles (lower and upper bounds of the box, respectively), lowest and highest data value within 1.5 times the interquartile range (lower and upper bounds of the whisker).

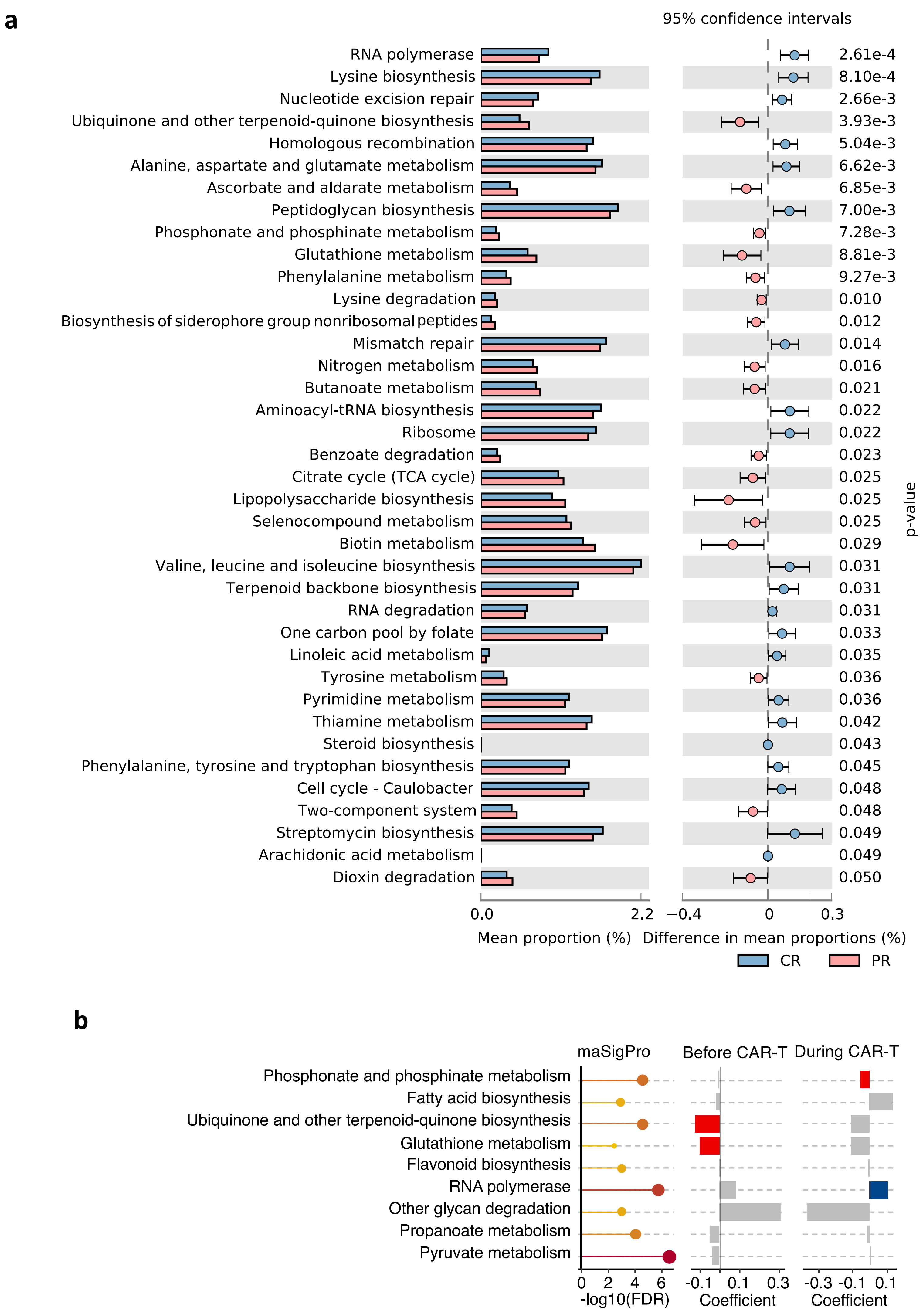

Supplementary Fig. 10. Differential KEGG pathways between CR and PR groups in the discovery MM cohort.

(a) Differential KEGG pathways in CR and PR groups measured by two-tailed Welch's t-test. Bar plot on left denotes mean proportion of each pathway in CR and PR groups. Dot plot on right depicts difference in mean proportion. Blue and red dots represent pathways enriched in CR and PR groups, respectively (n = 35). **(b)** Differential KEGG pathway analysis considering repeated measure design (n = 35). Bubble plot in the left represents p values by maSigPro. Barplots in the middle

and right show significances and coefficients by generalized linear-mixed models (GLMMs) before and after CAR-T infusion. Blue bars indicate significant enrichment in CR group while red bars indicate significant enrichment in PR group (FDR < 0.05).

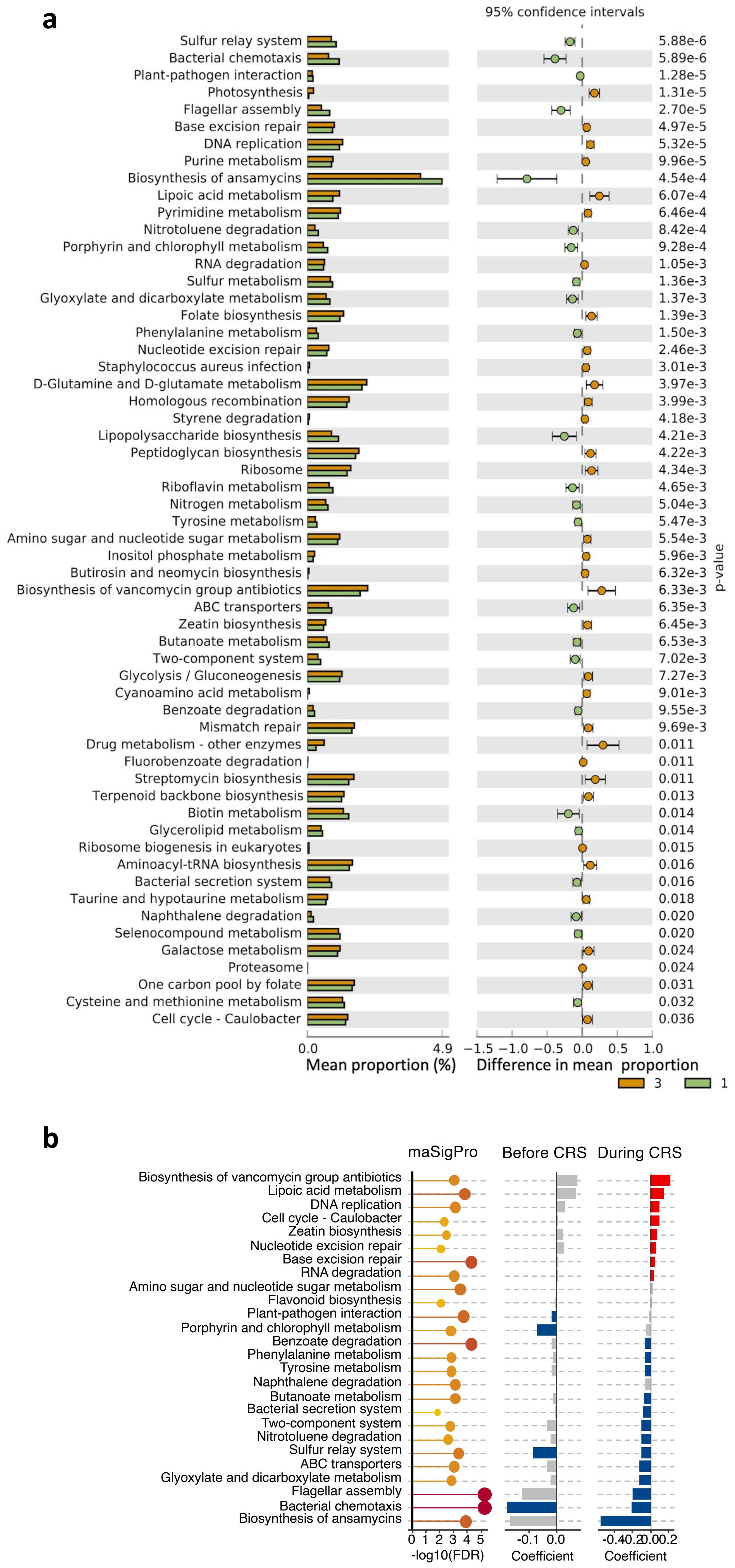

Supplementary Fig. 11. Differential KEGG pathways between CRS groups in the discovery MM cohort.

(a) Differential KEGG pathways between severe and mild CRS groups. Differential test was performed with two-tailed Welch’s t-test (n = 27). Bar plot on left denotes mean proportion of each pathway. Dot plot on right depicts difference in mean

proportion. **(b)** Differential KEGG pathway analysis considering repeated measure design (n = 27). Bubble plot in the left represents p values by maSigPro. Barplots in the middle and right show significances and coefficients by generalized linear-mixed models (GLMMs) before and after CAR-T infusion. Blue bars indicate negative correlation with CRS while red bars indicate positive correlation with CRS (FDR < 0.05).

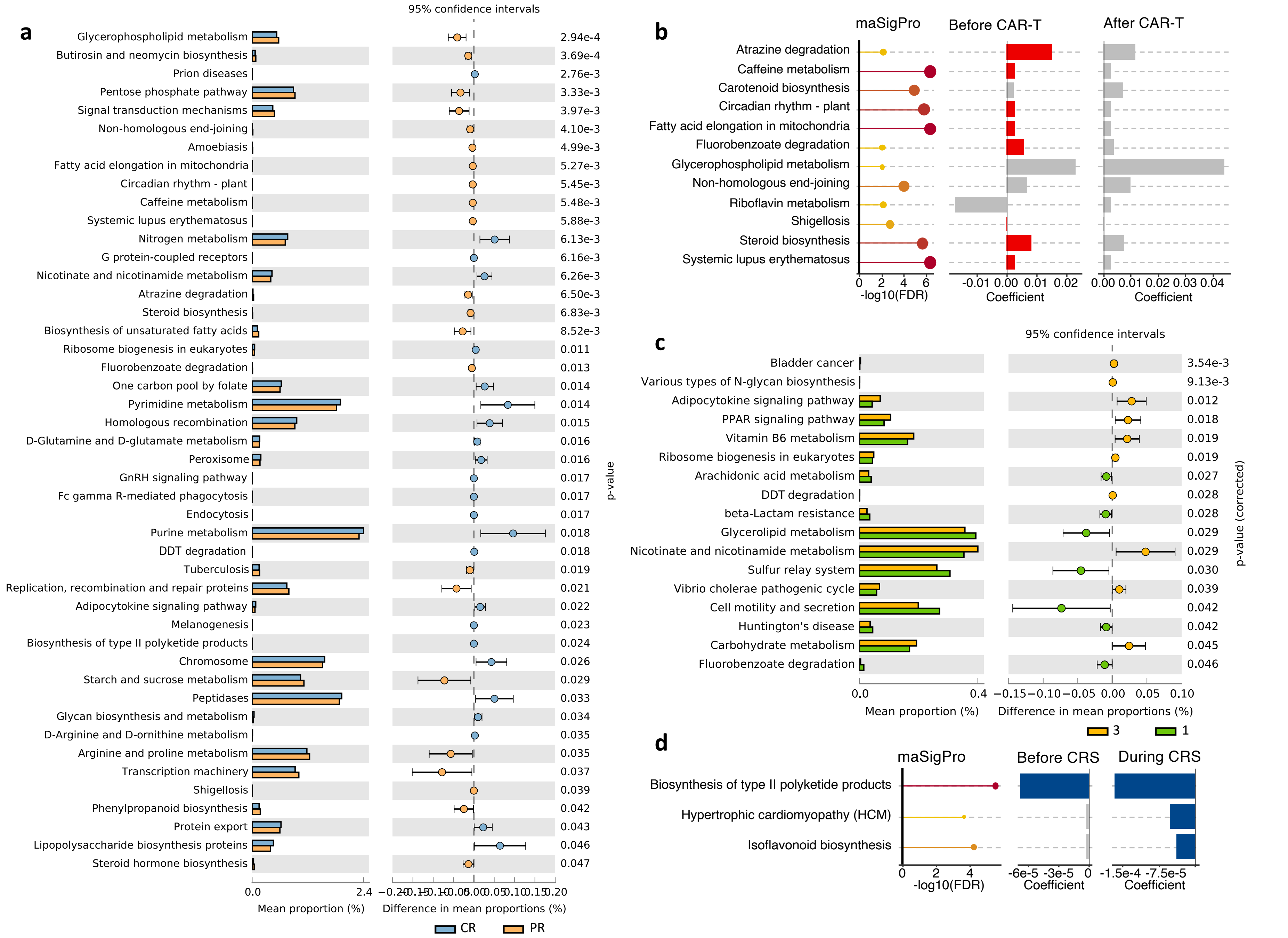

Supplementary Fig. 12. Differential analysis of KEGG pathways in the 38 MM validation cohort. (a) Differential KEGG pathways in CR and PR groups measured by two-tailed Welch's t-test. Bar plot on left denotes mean proportion of each pathway in CR and PR groups (n = 38). Dot plot on right depicts difference in mean proportion. Blue and red dots represent pathways enriched in CR and PR groups, respectively. **(b)** Differential KEGG pathway analysis considering repeated measure design (n = 10). Bubble plot in the left represents p values by maSigPro. Barplots in the middle and right show significances and coefficients by generalized linear-mixed models (GLMMs) before and after CAR-T infusion. Blue bars indicate significant enrichment in CR group while red bars indicate significant enrichment in PR group (FDR < 0.05). **(c)** Same as (**a**) for different CRS groups. **(d)** Same as (**b**) for different CRS groups.

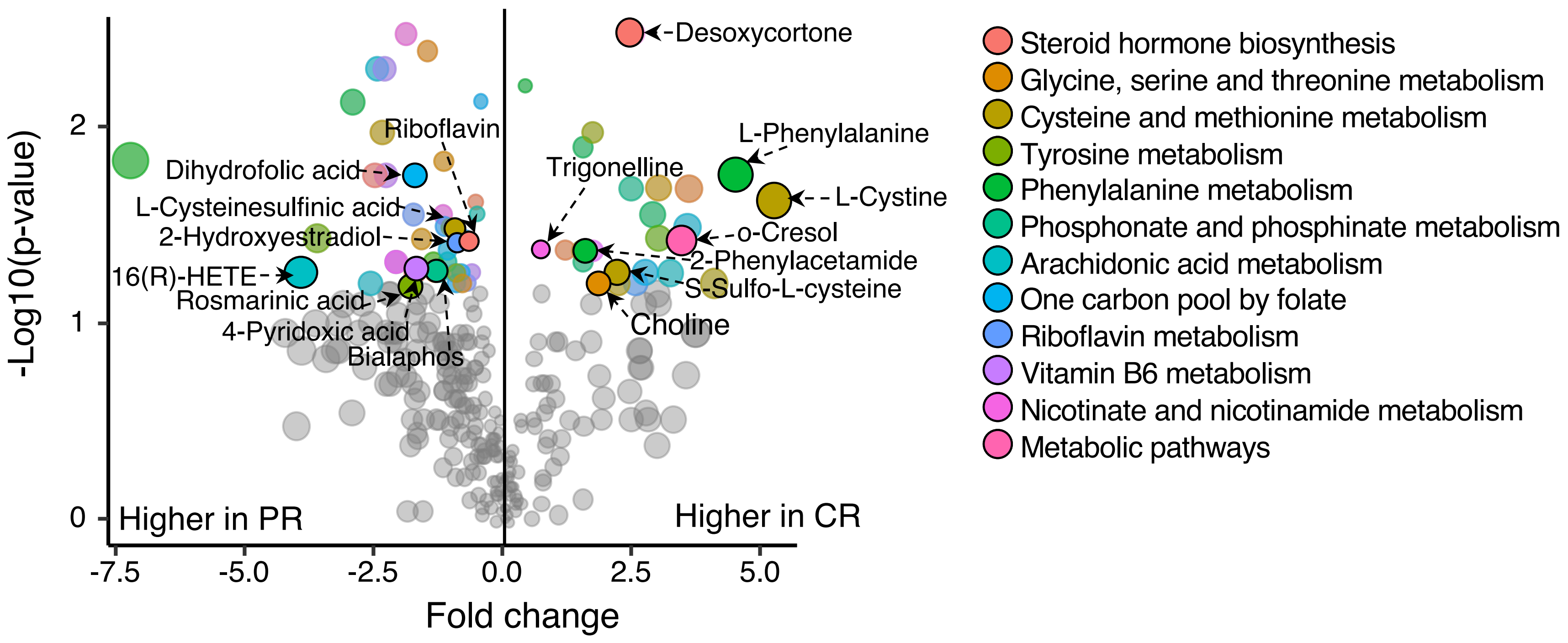

Supplementary Fig. 13. Differential metabolites between the CR and PR groups in the 38 MM validation cohort. Volcano plot shows fecal metabolites differentially abundant between the CR and PR groups (n = 31). Significance was assessed with two-tailed Wilcoxon rank-sum test. Size of points was proportional to log transformed p value. Metabolites with p value less than 0.05 were colored. Metabolites annotated to KEGG database were labelled in the plot. Legend in the rightside described KEGG pathways related to corresponding metabolites.

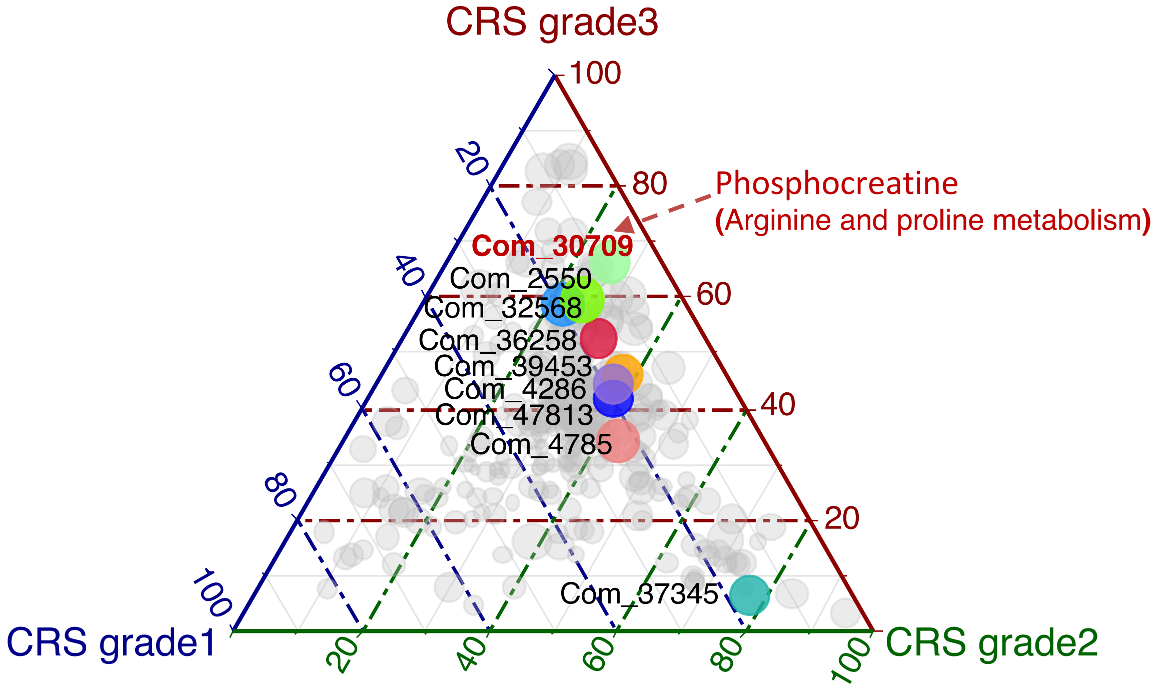

Supplementary Fig. 14. Differential metabolites between different CRS groups in the 38 MM validation cohort. Ternary phase diagram shows relative concentration of fecal metabolites among three CRS groups (n = 36). Significance was assessed with Kruskal-Wallis test. Metabolites with p value less than 0.05 were colored in the plot. Size of points was proportional to log transformed p value.

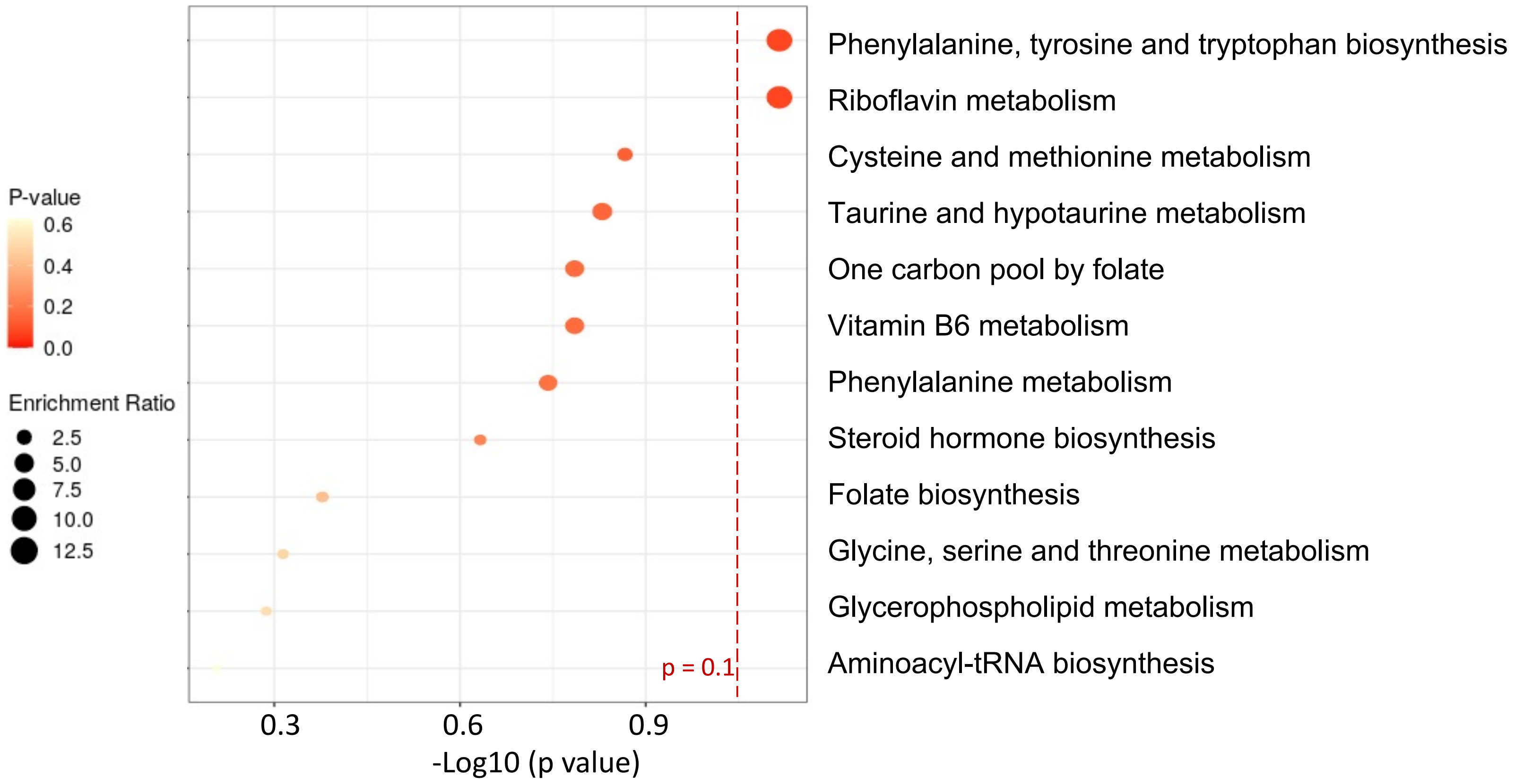

Supplementary Fig. 15. Pathway enrichment analysis for differentially abundant metabolites between CR and PR patients in 38 MM validation cohort. Bubble plot displays results for KEGG pathway enrichment analysis of differentially abundant metabolites between CR and PR patients in the MM validation cohort ($n = 31$, $p < 0.05$). The dashed red line indicates threshold of p-value = 0.1. Significance was assessed with Wilcoxon rank-sum test, with multiple testing correction.

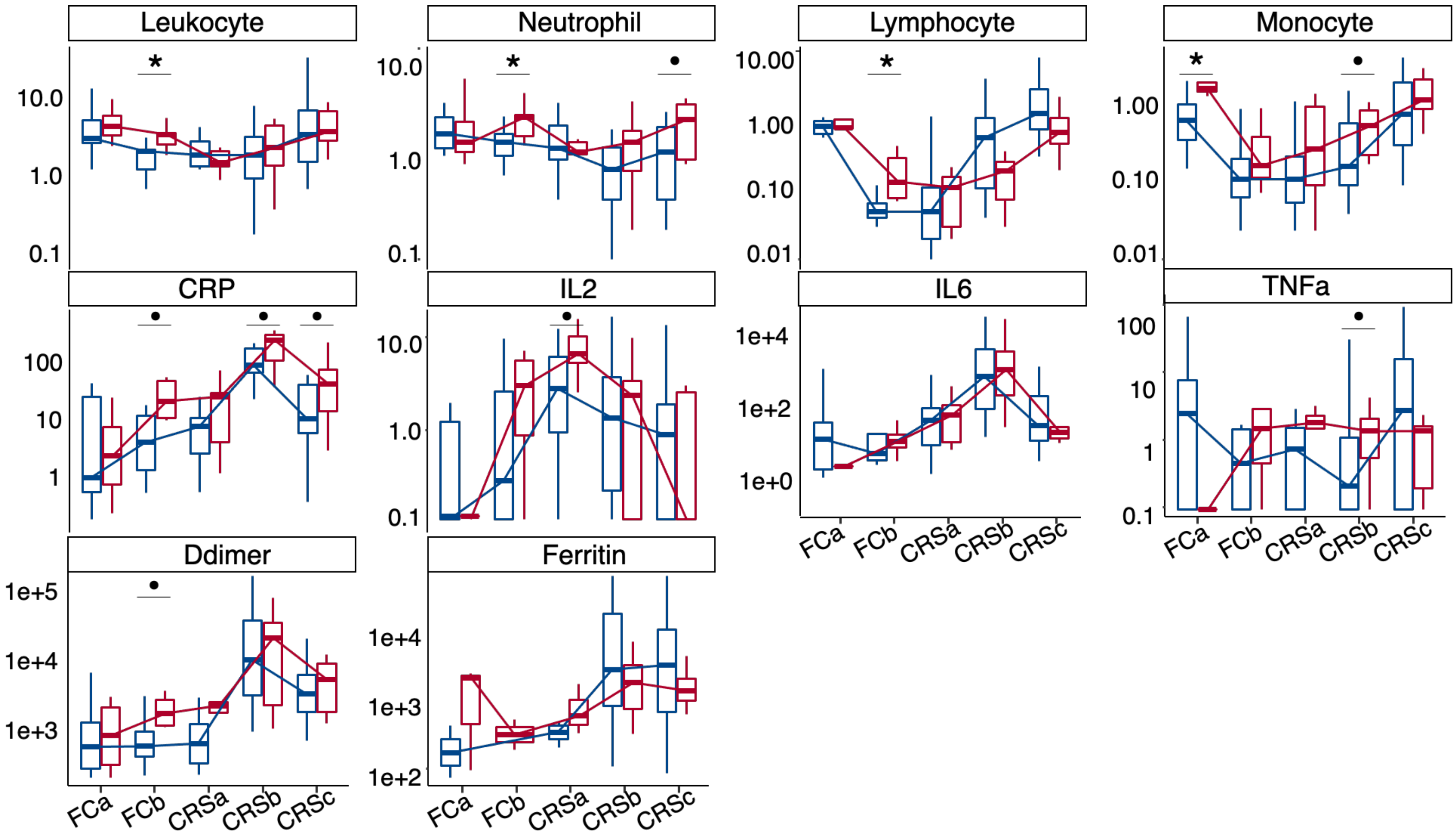

Supplementary Fig. 16. Differences of immune cells and inflammatory markers between CR and PR groups in the discovery MM cohort. Concentration of immune cells and inflammatory markers in different response groups across therapy stages (CR: blue; PR: red). Significance was assessed by Wilcoxon rank-sum test, with multiple testing correction (n = 35). While no significance passed the threshold of FDR < 0.05, we labelled differences with raw p values less than 0.1. For leukocyte: FCb, p = 0.01; neutrophil: FCb, CRSc, p = 0.042, 0.085, respectively; lymphocyte: FCb, p = 0.018; monocyte: FCa, CRSc, p = 0.023, 0.059, respectively; CRP: FCb, CRSb, CRSc, p = 0.093, 0.056, 0.070, respectively; IL2: CRSa, p = 0.086; TNFα: CRSb, p = 0.086; D-dimer: FCb, p = 0.066. Blue and red indicate CR and PR, respectively. Boxplots indicate the median (thick bar), first and third quartiles (lower and upper bounds of the box, respectively), lowest and highest data value within 1.5 times the interquartile range (lower and upper bounds of the whisker). Curves connected median value of different groups.

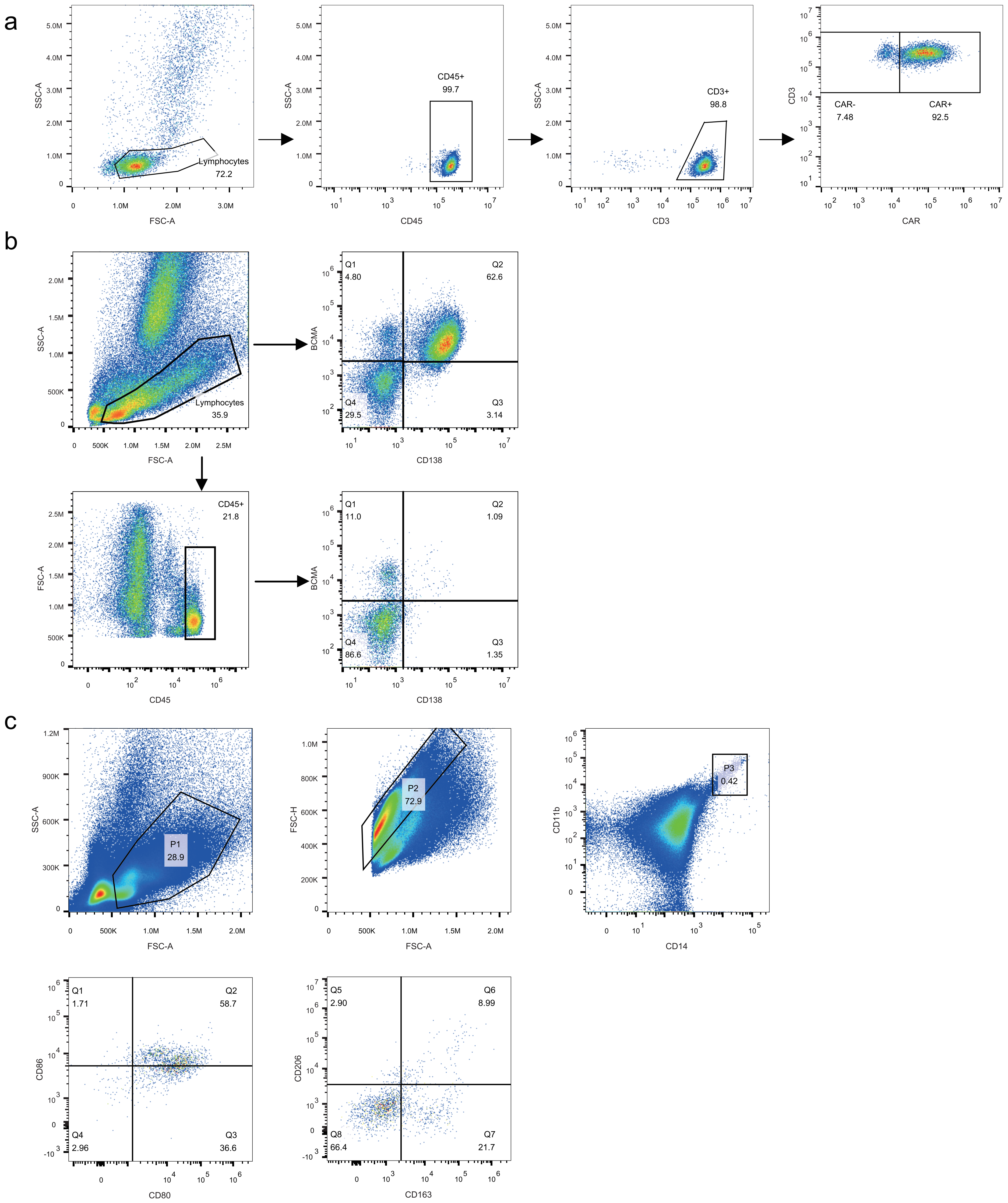

Supplementary Fig. 17. Flow cytometry gating strategies. **(a)** Flow cytometry gating strategy for CAR-T cell expansion test assays in figure 1e (left panel). **(b)** Flow cytometry gating strategy for tumor burden test assays in figure 1g (right panel). **(c)** Flow cytometry gating strategy for different macrophages phenotypes. The M1 phenotype was marked by CD80/CD86 and the M2 phenotype was marked by CD163/CD206.

Supplementary Table 1 Baseline characteristics of multiple myeloma patients included in final fecal microbiome analyses cohorts with different efficacy groups.

	CR N=24(%)	VGPR N=6(%)	PR N=11(%)	P
Age				0.412
Median	59.5	55.5	60	
Range	41-75	43-65	39-65	
Gender				1.0
Male	14(58.3)	4(66.7)	6(54.5)	
Female	10(41.7)	2(33.3)	5(45.5)	
Number of prior lines of therapy				0.197
Median	4	4.5	4	
Range	2-7	2-7	3-8	
CAR-T cell dose(×106/kg)				0.881
Median	4.65	4.03	4.4	
Range	1.2-6.9	2.5-6.2	1.3-5.8	
Autologous stem cell transplantation				0.158
No	17(70.8)	2(33.3)	5(45.5)	
Yes	7(29.2)	4(66.7)	6(54.5)	
Prior PI therapy				-
No	0(0)	0(0)	0(0)	
Yes	24(100)	6(100)	11(100)	
Prior IMiD therapy				0.341
No	1(4.2)	1(16.7)	0(0)	
Yes	23(95.8)	5(83.3)	11(100)	
Antibiotic use before or during treatment				
β -lactam	23(95.8)	6(100)	10(90.9)	0.663
Carbapenems	16(66.7)	3(50.0)	5(45.5)	0.504
Quinolone	15(62.5)	5(83.3)	5(45.5)	0.360
Aminoglycosides	0(0)	1(16.7)	0(0)	0.146
Macrolide	1(4.2)	0(0)	0(0)	1.0
Tetracyclines	2(8.3)	1(16.7)	1(9.1)	0.780
Cephalosporins	0(0)	1(16.7)	2(18.2)	0.064
Glycopeptides	1(4.2)	1(16.7)	3(27.3)	0.092

Notes: p-values calculated by Kruskal-Wallis test (age, number of prior lines of therapy, CAR-T cell dose), Chi-squared (gender, Autologous stem cell transplantation, Prior PI therapy, Prior IMiD therapy, Antibiotic use before or during treatment) and Fisher's exact test. P-value less than 0.05 was considered statistically significant. PI, Proteasome inhibitors (Bortezomib/Carfilzomib/Ixazomib). IMiD, immunomodulatory agent (Lenalidomide/Thalidomid/Pomalidomide).

Supplementary Table 2. Baseline characteristics of multiple myeloma patients included in final fecal microbiome analyses cohorts with different CRS grade groups.

	Grade 1 CRS N=8(%)	Grade 2 CRS N=16(%)	Grade 3 CRS N=19(%)	P
Age				0.571
Median	52.5	58.5	59	
Range	49-75	41-71	39-68	
Gender				0.567
Male	6(75)	8(50)	10(52.6)	
Female	2(25)	8(50)	9(47.4)	
Number of prior lines of therapy				0.081
Median	3	4.5	4	
Range	2-4	2-8	2-7	
CAR-T cell dose(×106/kg)				0.349
Median	3.73	4.96	4.4	
Range	2.7-5.8	2.4-6.9	1.2-5.3	
Autologous stem cell transplantation				0.920
No	5(62.5)	9(56.3)	12(63.2)	
Yes	3(37.5)	7(43.7)	7(36.8)	
Prior PI therapy				-
No	0(0)	0(0)	0(0)	
Yes	8(100)	16(100)	19(100)	
Prior IMiD therapy				0.306
No	1(12.5)	1(6.3)	0(0)	
Yes	7(87.5)	15(93.7)	19(100)	
Antibiotic use before or during treatment				
β -lactam	8(100)	14(87.5)	19(100)	0.164
Carbapenems	4(50)	7(43.8)	15(78.9)	0.096
Quinolone	4(50)	7(43.8)	15(78.9)	0.096
Aminoglycosides	0(0)	1(6.3)	0(0)	0.558
Macrolide	0(0)	0(0)	1(5.3)	1
Tetracyclines	0(0)	0(0)	4(21.1)	0.09
Cephalosporins	0(0)	2(12.5)	1(5.3)	0.581
Glycopeptides	0(0)	2(12.5)	4(21.1)	0.457

p-values calculated by Kruskal-Wallis test (age, Number of prior lines of therapy, CAR-T cell dose), Chi-squared (gender, Autologous stem cell transplantation, Prior PI therapy, Prior IMiD therapy, Antibiotic use before or during treatment) and Fisher's exact test. P-value less than 0.05 was considered statistically significant. PI, Proteasome inhibitors (Bortezomib/Carfilzomib/Ixazomib). IMiD, immunomodulatory agent (Lenalidomide/Thalidomid/Pomalidomide).

Supplementary Table 3. Demographic and clinical characteristics of B-ALL and B-NHL patients included in final fecal microbiome analyses cohorts.

	B-ALL N=23 (%)	B-NHL N=12 (%)
Age		
Median	42	58
Range	14-67	25-71
Gender		
Male	11 (47.8)	6 (50)
Female	12 (52.2)	6 (50)
Number of prior lines of therapy		
Median	3	3
Range	2-9	2-5
CAR-T cell dose(×106/kg)		
Median	5.92	5.79
Range	2.39-12.59	4.84-8.98
Cytokine release syndrome		
Grade 1	4 (17.4)	4 (33.3)
Grade 2	12 (52.2)	8 (66.7)
Grade 3	7 (30.4)	0
Grade 4	0	0
Grade 5	0	0
Response		
CR	21 (91.3)	9 (75)
PR	0	2 (16.7)
NR	2 (8.7)	1 (8.3)

Supplementary Methods

Assessment of differentiated macrophages in the longitudinal analysis

Peripheral blood mononuclear cells (PBMCs) were collected before and after CAR-T cells infusion. Macrophages surfaces markers were detected by flow cytometry for evaluation of different phenotypes. The *M1* phenotype was marked by CD80/CD86 (BioLegend, 305218/BioLegend, 305414) and the *M2* phentype was marked by CD163/CD206 (BioLgend, 333610/eBioscience, 12-2069-42).

LC-MS/MS Analysis

LC-MS/MS analyses were performed using a Vanquish UHPLC system (ThermoFisher, Germany) coupled with an Orbitrap Q Exactive^{TM}HF-X Mass Spectrometer (Thermo Fisher，Germany). Samples were injected onto a Hypesil Gold column (100×2.1 mm, 1.9μm) using a 17-min linear gradient at a flow rate of 0.2mL/min. The eluents for the positive polarity mode were eluent A (0.1% FA in Water) and eluent B (Methanol).The eluents for the negative polarity mode were eluent A (5 mM ammonium acetate, pH 9.0) and eluent B (Methanol).The solvent gradient was set as follows: 2% B, 1.5 min; 2-85% B, 3 min; 100% B, 10 min; 100-2% B, 10.1 min；2% B, 12 min. Q Exactive^{TM} HF Mass Spectrometer was operated in positive/negative polarity mode with spray voltage of 3.2 kV, capillary temperature of 320°C, sheath gas flow rate of 40 arb and aux gas flow rate of 10 arb, Funnel RF level of 40, Aux gas heater temperature of 350°C.
