# Supplementary material for: CAR-T cell therapy-related cytokine release syndrome and therapeutic response is modulated by the gut microbiome in hematologic malignancies
Source: Nat Commun. 2022 Sep 9;13:5313. doi: 10.1038/s41467-022-32960-3 (PMC9461447; doi:10.1038/s41467-022-32960-3)
Supplement: Supplementary file 3 — Description of Additional Supplementary Files [file 41467_2022_32960_MOESM3_ESM.pdf]

Description of Additional Supplementary Files

File Name: Supplementary Data 1
Description: Number of samples collected at each stage for each patient in MM, ALL and NHL samples

File Name: Supplementary Data 2
Description: Sequencing depth for each patient in MM, NHL and ALL samples

File Name: Supplementary Data 3
Description: Taxonomies correlated with therapy stages identified by longitudinal analysis in Qiime2.

File Name: Supplementary Data 4
Description: **Taxonomies correlated with therapy stages identified by time-course differential analysis in maSigPro in MM patients.** Negative binominal regressive model was applied followed by multiple testing correction.

File Name: Supplementary Data 5
Description: **OTUs identified as having differential time-course patterns between CR and PR patients by maSigPro in MM patients.** Negative binominal regressive model with multiple testing correction was used.

File Name: Supplementary Data 6
Description: **Genera identified as having differential time-course patterns between CR and PR groups in MM patients.** Negative binominal regressive model with multiple testing correction was applied for maSigPro to test significant differences between CR and PR groups. Linear discriminant analysis (LDA) analysis and generalized linear-mixed model was implemented followed by multiple testing correction to identify genera with different abundances between CR and PR groups before and after CAR-T infusion.

File Name: Supplementary Data 7
Description: **maSigPro identified OTUs having differential time-course patterns between individuals with severe (CRS = 3) and mild (CRS = 1) cytokine release syndrome among the MM patients.** Negative binominal regressive model with multiple testing correction was used.

File Name: Supplementary Data 8
Description: **maSigPro identified OTUs having differential time-course patterns between individuals with severe (CRS = 3) and moderate (CRS ≤ 2) cytokine release syndrome among the MM patients.** Negative binominal regressive model with multiple testing correction was used.

File Name: Supplementary Data 9
Description: **Genera identified as having differential time-course patterns between individuals with sever (CRS = 3) and mild (CRS = 1) cytokine release syndrome among MM patients.** Negative binominal regressive model with multiple testing correction was applied for maSigPro to test significant differences between sever and mild groups. Linear discriminant analysis (LDA) analysis and generalized linear-mixed model was implemented followed by multiple testing correction to identify genera with different abundances between sever and mild groups before and after CAR-T infusion.
