# Supplementary material for: CAR-T cell therapy-related cytokine release syndrome and therapeutic response is modulated by the gut microbiome in hematologic malignancies
Source: Nat Commun. 2022 Sep 9;13:5313. doi: 10.1038/s41467-022-32960-3 (PMC9461447; doi:10.1038/s41467-022-32960-3)
Supplement: Supplementary file 13 — Reporting Summary [file 41467_2022_32960_MOESM13_ESM.pdf]

nature portfolio

Corresponding author(s): He Huang

Last updated by author(s): 2022-7-20

Reporting Summary

Nature Portfolio wishes to improve the reproducibility of the work that we publish. This form provides structure for consistency and transparency in reporting. For further information on Nature Portfolio policies, see our Editorial Policies and the Editorial Policy Checklist.

Statistics

For all statistical analyses, confirm that the following items are present in the figure legend, table legend, main text, or Methods section.

n/a	Confirmed	
☐	☒	The exact sample size (*n*) for each experimental group/condition, given as a discrete number and unit of measurement
☐	☒	A statement on whether measurements were taken from distinct samples or whether the same sample was measured repeatedly
☐	☒	The statistical test(s) used AND whether they are one- or two-sided<br>*Only common tests should be described solely by name; describe more complex techniques in the Methods section.*
☐	☒	A description of all covariates tested
☐	☒	A description of any assumptions or corrections, such as tests of normality and adjustment for multiple comparisons
☐	☒	A full description of the statistical parameters including central tendency (e.g. means) or other basic estimates (e.g. regression coefficient) AND variation (e.g. standard deviation) or associated estimates of uncertainty (e.g. confidence intervals)
☐	☒	For null hypothesis testing, the test statistic (e.g. *F*, *t*, *r*) with confidence intervals, effect sizes, degrees of freedom and *P* value noted<br>*Give P values as exact values whenever suitable.*
☒	☐	For Bayesian analysis, information on the choice of priors and Markov chain Monte Carlo settings
☒	☐	For hierarchical and complex designs, identification of the appropriate level for tests and full reporting of outcomes
☒	☐	Estimates of effect sizes (e.g. Cohen's *d*, Pearson's *r*), indicating how they were calculated

Our web collection on statistics for biologists contains articles on many of the points above.

Software and code

Policy information about availability of computer code

Data collection	No software used
Data analysis	SPSS 23.0; R (v. 3.6.2); Qiime2 (v. 2020.2); VSEARCH (v. 2.4.4); PICRUSt2 (v.2.3.0-b); The LAD effect size (LEfSe, v.1.1); Cytoscape (v.3.9.0);R packages: vegan (v. 2.5-6), caret (v. 6.0-85), maSigPro (v.1.58.0), recursive feature elimination (RFE) algorithm within stats (v.3.6.2), pROC (v.1.16.1), survminer (v.0.4.7), rmcorr (v.0.4.5). FlowJo (V10).

For manuscripts utilizing custom algorithms or software that are central to the research but not yet described in published literature, software must be made available to editors and reviewers. We strongly encourage code deposition in a community repository (e.g. GitHub). See the Nature Portfolio guidelines for submitting code & software for further information.

Data

Policy information about availability of data

All manuscripts must include a data availability statement. This statement should provide the following information, where applicable:

- Accession codes, unique identifiers, or web links for publicly available datasets
- A description of any restrictions on data availability
- For clinical datasets or third party data, please ensure that the statement adheres to our policy

Data and materials availability: The raw sequence reads generated in this study have been deposited in the Sequence Read Archive (SRA) of the NCBI under accession number PRJNA813944. All softwares used for analyses are publicly available for download. Greengenes database can be accessed from https://greengenes.secondgenome.com/?prefix=downloads/greengenes_database/gg_13_5/. KEGG database can be downloaded from https://www.kegg.jp/kegg-bin/download_htext?htext=hsa00001&format=htext&filedir=kegg/brite/hsa. Pre-trained Naive Bayes classifier trained on the Greengenes 13_8 99% OTU database can be accessed from https://data.qiime2.org/2020.2/common/gg-13-8-99-515-806-nb-classifier.qza.

Field-specific reporting

Please select the one below that is the best fit for your research. If you are not sure, read the appropriate sections before making your selection.

☒ Life sciences ☐ Behavioural & social sciences ☐ Ecological, evolutionary & environmental sciences

For a reference copy of the document with all sections, see nature.com/documents/nr-reporting-summary-flat.pdf

Life sciences study design

All studies must disclose on these points even when the disclosure is negative.

Sample size	Ninety-nine patients with relapsed/refractory multiple myeloma (r/r MM) were included in this trial. Microbiome samples were not available from 12 patients and 16S sequencing depth was not sufficient for analysis on 6 patients. Finally, a total of 81 patients with r/r MM was included for gut microbiome analysis, which included 43 patients for experiment group and 38 patients for validation group. The fecal sample collection occurred from 2018 to2021. The sample size this cohort was determined based upon the number of patients at Bone Marrow Transplantation Center, The First Affiliated Hospital, School of Medicine, Zhejiang University who were treated with BCMA targeted CART cells and consented to fecal collection.<br>A sample size calculation was not performed to determine the sample size. As noted above, the sample size was determined based upon the number of relapsed/refractory multiple myeloma (r/r MM) patients treated at Bone Marrow Transplantation Center, The First Affiliated Hospital, School of Medicine, Zhejiang University treated with BCMA CART cells from 2018 to 2021.
Data exclusions	Microbiome samples were not available from 12 patients and 16S sequencing depth was not sufficient for analysis on 6 patients.
Replication	we included 38 MM patients as validation group. Not all but most attempts at replication (validation) were successful.
Randomization	This study does not involve an intervention. So, the patients were not randomized.
Blinding	This study does not involve an intervention and it does not compare treatments. So, the there was no blinding.

Reporting for specific materials, systems and methods

We require information from authors about some types of materials, experimental systems and methods used in many studies. Here, indicate whether each material, system or method listed is relevant to your study. If you are not sure if a list item applies to your research, read the appropriate section before selecting a response.

Materials & experimental systems

n/a	Involved in the study	
☐	☒	Antibodies
☒	☐	Eukaryotic cell lines
☒	☐	Palaeontology and archaeology
☒	☐	Animals and other organisms
☐	☒	Human research participants
☐	☒	Clinical data
☒	☐	Dual use research of concern

Methods

n/a	Involved in the study	
☒	☐	ChIP-seq
☐	☒	Flow cytometry
☒	☐	MRI-based neuroimaging

Antibodies

Antibodies used	BV510 Mouse anti-human CD45(Clone:HI30, Cat:563204,Lot:1026875) , BD Biosciences; APC anti-human CD3(Clone:UCHT1, Cat:300439, Lot:B333984), Biolegend; PE anti-human CD269(BCMA)(Clone:19F2, Cat:357504,Lot:B325846), Biolegend; FITC anti-human CD138(Clone:MI15,Cat:356508,Lot: B270191), Biolegend; APC anti-humanCD45(Clone:2D1, Cat:368512,Lot: B263921), Biolegend; PE-Cy7 anti-human CD3(Clone:UCHT1, REF:25-0038-42,Lot: 2391882), eBioscience; PerCP-Cy5.5 anti-human CD4(Clone:RPA-T4, Cat:560650,Lot: 0195587), BD Biosciences; PE anti-human CD8(Clone:RPA-T8, Cat:301051,Lot: B292215), Biolegend; Biotin-SP-conjugated Affinipure F(ab')2 Fragment Goat Anti-Mouse IgG, F(ab')2 Fragment Specific, Jackson (Code number:115-066-006,Lot number: 155159); FITC Streptavidin(cat:405202,Lot:B319211), Biolegend; BV605 anti-human CD14, (Biolegend, Clone:M5E2,cat:301834,Lot:B337744);BV421 anti-human CD11b(Biolegend, Clone:ICRF44,cat:301324,Lot:B346982);PE anti-human CD206(ebioscience, Clone:19.2,Lot:2344972,REF:12-2069-42); APC anti-human CD163(Biolegend, Clone:GHI/61,cat:333610,Lot:B338712); PE-cy7 anti-human CD80(Biolegend, Clone:2D10,cat:305218.Lot:B341123);AF488 anti-human CD86(Biolegend, Clone:IT2.2,cat:305414,Lot:B320841).
Validation	Used antibodies were titrated or used as recommended by the manufacturer. All the antibodies are validated for use in flow cytometry. Species validation and antibody data are available on the manufacturer's website. All used antibodies are commercially available. BV510 Mouse anti-human CD45(Clone:HI30, Cat:563204,Lot:1026875) ：https://www.bdbiosciences.com/en-us/

products/reagents/flow-cytometry-reagents/research-reagents/single-color-antibodies-ruo/bv510-mouse-anti-human-cd45.563204
APC anti-human CD3(Clone:UCHT1, Cat:300439, Lot:B333984), Biolegend：https://www.biolegend.com/en-us/products/apc-anti-human-cd3-antibody-861
PE anti-human CD269(BCMA)(Clone:19F2, Cat:357504,Lot:B325846), Biolegend：https://www.biolegend.com/en-us/products/pe-anti-human-cd269-bcma-antibody-8446
FITC anti-human CD138(Clone:MI15,Cat:356508,Lot: B270191), Biolegend：https://www.biolegend.com/en-us/products/fitc-anti-human-cd138-syndecan-1-antibody-8406
APC anti-humanCD45(Clone:2D1, Cat:368512,Lot: B263921), Biolegend：https://www.biolegend.com/en-us/products/apc-anti-human-cd45-antibody-12397
PE-Cy7 anti-human CD3(Clone:UCHT1, REF:25-0038-42,Lot: 2391882), eBioscience：https://www.thermofisher.cn/cn/zh/antibody/product/CD3-Antibody-clone-UCHT1-Monoclonal/25-0038-42
PerCP-Cy5.5 anti-human CD4(Clone:RPA-T4, Cat:560650,Lot: 0195587), BD Biosciences：https://www.bdbiosciences.com/content/dam/bdb/products/global/reagents/flow-cytometry-reagents/research-reagents/single-color-antibodies-ruo/560650_base/pdf/560650.pdf
PE anti-human CD8(Clone:RPA-T8, Cat:301051,Lot: B292215), Biolegend：https://www.biolegend.com/en-us/products/pe-anti-human-cd8a-antibody-836
Biotin-SP-conjugated Affinipure F(ab')2 Fragment Goat Anti-Mouse IgG, F(ab')2 Fragment Specific, Jackson (Code number:115-066-006,Lot number: 155159)：https://www.jacksonimmuno.com/catalog/products/115-066-006
FITC Streptavidin(cat:405202,Lot:B319211), Biolegend：https://www.biolegend.com/en-us/products/fitc-streptavidin-1473
BV605 anti-human CD14,(Biolegend, Clone:M5E2,cat:301834,Lot:B337744)：https://www.biolegend.com/en-us/products/brilliant-violet-605-anti-human-cd14-antibody-7653
BV421 anti-human CD11b(Biolegend, Clone:ICRF44,cat:301324,Lot:B346982)：https://www.biolegend.com/en-us/products/brilliant-violet-421-anti-human-cd11b-antibody-7325
PE anti-human CD206(ebioscience, Clone:19.2,Lot:2344972,REF:12-2069-42)：https://www.thermofisher.cn/cn/zh/antibody/product/CD206-MMR-Antibody-clone-19-2-Monoclonal/12-2069-42
APC anti-human CD163(Biolegend, Clone:GHI/61,cat:333610,Lot:B338712)：https://www.biolegend.com/en-us/products/apc-anti-human-cd163-antibody-6276
PE-cy7 anti-human CD80(Biolegend, Clone:2D10,cat:305218.Lot:B341123)：https://www.biolegend.com/en-us/products/pe-cyanine7-anti-human-cd80-antibody-6174
AF488 anti-human CD86(Biolegend, Clone:IT2.2,cat:305414,Lot:B320841).：https://www.biolegend.com/en-us/products/alexa-fluor-488-anti-human-cd86-antibody-3355

Human research participants

Policy information about studies involving human research participants

Population characteristics

The median age of the MM patients was 59 (range 39–75) years, and 55.8% were male (Table 1). The median number of prior lines of therapy was 4 (range 2–8), with all receiving proteasome inhibitor therapy and 95.3% immunomodulatory agents. At enrollment, 39.5% had received autologous stem cell transplantation, and 55.8% had extramedullary disease(s).

Recruitment

All patients provided written informed consent for participation in accordance with the guidelines of the Declaration of Helsinki and signed agreement for collection and analysis of microbiome samples. Patient inclusion criteria were: (1) age < 75 years; (2) relapsed or refractory BCMA–positive MM before CAR-T cell treatment; and (3) expected survival > 12 weeks and adequate performance status and organ function to tolerate treatment. Exclusion criteria were: (1) pregnancy or lactation; (2) having received systemic (except inhaled) steroids in the previous 2 weeks or gene therapies; (3) having medical conditions such as severe mental illness, clinically significant cardiovascular disease, severe renal or hepatic dysfunction, or active infection; and (4) any conditions that might increase treatment risks.
There was not any self-selection or other bias present in the recruitment of the patients to either of the cohorts noted.

Ethics oversight

The study was approved by the Institutional Review Board of the First Affiliated Hospital, School of Medicine, Zhejiang University

Note that full information on the approval of the study protocol must also be provided in the manuscript.

Clinical data

Policy information about clinical studies

All manuscripts should comply with the ICMJE guidelines for publication of clinical research and a completed CONSORT checklist must be included with all submissions.

Clinical trial registration

The study was registered in the Chinese Clinical Trial Registry (ChiCTR1800017404). All patients provided written informed consent for participation in accordance with the guidelines of the Declaration of Helsinki and signed agreement for collection and analysis of microbiome samples.

Study protocol	The study was registered in the Chinese Clinical Trial Registry with identifier ChiCTR1800017404.
Data collection	clinical data collection were carried from July 1st 2018 to September 30th 2021, at Bone Marrow Transplantation Center, The First Affiliated Hospital, School of Medicine, Zhejiang University, Hangzhou, China;
Outcomes	We assessed the BCMA CAR-T cell safety and efficacy and valuated the association between gut microbiome with clinical outcomes after BCMA CAR-T cell treatment. The clinical outcomes that we assessed include the following: clinical response(complete response (CR), very good partial response (VGPR), or partial response (PR)) in the third month after CAR-T treatment, progression-free survival(PFS) and cytokine release syndrome (CRS).

Flow Cytometry

Plots

Confirm that:

☒ The axis labels state the marker and fluorochrome used (e.g. CD4-FITC).

☒ The axis scales are clearly visible. Include numbers along axes only for bottom left plot of group (a 'group' is an analysis of identical markers).

☒ All plots are contour plots with outliers or pseudocolor plots.

☒ A numerical value for number of cells or percentage (with statistics) is provided.

Methodology

Sample preparation	Serial blood samples were collected in BD Vacutainer K2EDTA tubes (BD Biosciences) before and after CAR-T cell infusion. All blood samples were stored at 4°C until centrifugation at 5000 rpm for 6 min to Assess the serum cytokine concentrations and CAR-T cell expansion and persistence.
Instrument	CytoFLEX
Software	FlowJo v10.1
Cell population abundance	no sorting was performed.
Gating strategy	Gating strategies are provided in the Supplementary information.

☒ Tick this box to confirm that a figure exemplifying the gating strategy is provided in the Supplementary Information.
